# Microbial communities colonising plastics during transition from the wastewater treatment plant to marine waters

**DOI:** 10.1186/s40793-024-00569-2

**Published:** 2024-04-29

**Authors:** Constance L. Tulloch, Rafael Bargiela, Gwion B. Williams, Tatyana N. Chernikova, Benjamin M. Cotterell, Elizabeth M. H. Wellington, Joseph Christie-Oleza, David N. Thomas, Davey L. Jones, Peter N. Golyshin

**Affiliations:** 1 Centre for Environmental Biotechnology, School of Environmental and Natural Sciences, Bangor University, Bangor, LL57 2UW UK; 2 School of Life Sciences, University of Warwick, Coventry, CV4 7AL UK; 3 Department of Biology, University of the Balearic Islands, 07122 Palma, Spain; 4 Faculty of Biological and Environmental Sciences, University of Helsinki, 00014 Helsinki, Finland

## Abstract

**Background:**

Plastics pollution and antimicrobial resistance (AMR) are two major environmental threats, but potential connections between plastic associated biofilms, the ‘plastisphere’, and dissemination of AMR genes are not well explored.

**Results:**

We conducted mesocosm experiments tracking microbial community changes on plastic surfaces transitioning from wastewater effluent to marine environments over 16 weeks. Commonly used plastics, polypropylene (PP), high density polyethylene (HDPE), low density polyethylene (LDPE) and polyethylene terephthalate (PET) incubated in wastewater effluent, river water, estuarine water, and in the seawater for 16 weeks, were analysed via 16S rRNA gene amplicon and shotgun metagenome sequencing. Within one week, plastic-colonizing communities shifted from wastewater effluent-associated microorganisms to marine taxa, some members of which (e.g. *Oleibacter-Thalassolituus* and *Sphingomonas* spp., on PET, *Alcanivoracaceae* on PET and PP, or *Oleiphilaceae,* on all polymers), were selectively enriched from levels undetectable in the starting communities. Remarkably, microbial biofilms were also susceptible to parasitism, with *Saprospiraceae* feeding on biofilms at late colonisation stages (from week 6 onwards), while *Bdellovibrionaceae* were prominently present on HDPE from week 2 and LDPE from day 1. Relative AMR gene abundance declined over time, and plastics did not become enriched for key AMR genes after wastewater exposure.

**Conclusion:**

Although some resistance genes occurred during the mesocosm transition on plastic substrata, those originated from the seawater organisms. Overall, plastic surfaces incubated in wastewater did not act as hotspots for AMR proliferation in simulated marine environments.

**Supplementary Information:**

The online version contains supplementary material available at 10.1186/s40793-024-00569-2.

## Introduction

Antimicrobial resistance (AMR) is a global public health threat predominantly driven by poor administration of medications to treat bacterial, viral, fungal or parasitic infections in humans and animals [1]. Secondary causes of increasing AMR incidence include agricultural use, poor sanitation, restricted access to clean water and the discharge of human derived wastewater to the environment [1, 2]. Conventional wastewater treatment plants (WWTP) can significantly reduce the antibiotic and AMR load of wastewater via processes such as sedimentation, filtration, and disinfection, however, they often fail to eliminate all AMR carrying bacteria [3]. It has been hypothesised that microplastics contained within wastewater can act as hubs and effective carriers of microbial pathogens and their AMR-genes (ARGs) and that this may facilitate their persistence during passage through the WWTP [4, 5]. It has been suggested that colonisation of plastic surfaces by AMR carrying organisms may promote their dispersal and persistence when WWTP effluent is discharged into the wider environment [4–7].

The ubiquity of plastic pollution is well established with up to 80% of marine debris being present as either nano-, micro-, and macro-plastics [8, 9]. Further, ca. 80% of this is thought to originate from the terrestrial environment, with the remainder being from sea-based sources [8–10]. The primary routes by which plastics enter the marine zone include littering, road-runoff, landfill leachate and wastewater discharges [11, 12]. The majority of macro- and microplastics (MP) in urban wastewater are removed following preliminary and primary wastewater treatment by surface skimming or grit settling, depending on particle buoyancy, and end up in the biosolids fraction which is typically incinerated, sent to landfill or applied to the land [4, 6, 8, 13]. However, WWTPs do not utilise specific plastic removal technologies and consequently, a small but significant fraction of the total plastic load is discharged to the environment after treatment [4, 5, 11, 13]. In addition, during heavy rainfall events, untreated wastewater with a high plastic load is often discharged directly into the environment via combined sewer overflows (CSOs; [14]). The release of wastewater from CSOs is also predicted to increase in the future in response to climate change and increasing urbanisation, potentially leading to greater discharges of plastics and microorganisms harbouring ARG into freshwaters and the marine environment [15].

Weathered plastic has an inherent pit forming nature, which increases its surface area and suitability for biofilm formation [7]. Further, their hydrophobic surface characteristics make environmental plastics a prime site for microbial colonisation [7, 16–18]. By acting as a vector, plastic may transport harmful pathogenic microorganisms and invasive species to new environments, protecting them from harsh oceanic conditions (e.g., UV irradiation, salinity) while also providing a surface for horizontal gene transfer (HGT) to take place—therefore increasing the incidence of AMR among colonisers [17].

The plastisphere, a term coined by Zettler et al. [17], describes the dynamic ecosystem that forms on plastic in aquatic environments. Previous research linking the plastisphere and AMR has found both non-pathogenic microorganisms (e.g., *Acidovorax, Sphingomonas, Rhodobacter* and *Aquabacterium*) and opportunistic pathogens (e.g., *Xanthomonas*, *Klebsiella* and *Pseudomonas* spp.) on the surface of plastics recovered from rivers polluted with wastewater [7, 19]. Potentially pathogenic *Pseudomonas* spp*.* have also been detected exclusively on riverine plastic [16, 20]. Additionally, bacteria commonly recovered from WWTP effluent such as, *Aeromonas*, *Bacillus*, *Pseudomonas* spp. and *Comamonas* spp. have been detected on polyethylene terephthalate (PET) and low-density polyethylene (LDPE) downstream of the WWTP [21, 22]. *Vibrio* spp., possibly the most researched bacteria regarding the plastisphere due to its implication in human infection, were also detected on plastics present in polluted rivers [7]. Interestingly, microbial community composition on plastic appears to be more dependent on local conditions like salinity, light and surface biofilms rather than intrinsic plastic properties [18, 21, 23–26].

Antimicrobial compounds in wastewater act as selection pressures encouraging ARG expression and HGT [16]. Munk and colleagues [27] identified 13 universal ARG from sewage from 101 countries including tetracycline resistance genes *tetA*, *tetC* and *tetW*, sulfonamide resistance genes *sul1* and *sul2*, as well as macrolide resistance genes *mphE* and *msrE*. With plastic acting as a vector for AMR bacteria, the number of globally universal ARG detected will continue to increase.

The aim of this study was to use a series of mesocosms to simulate plastic waste transport from the wastewater treatment plant to the marine zone, by successively exposing different types of plastic to wastewater effluent, freshwater, estuarine and seawater. We tracked the changes in bacterial community structure on the surface of the plastics from the early stages of colonisation up to 16 weeks, simulating the residence times taken in each water type. SSU rRNA V4 region amplicon sequencing and the whole genome sequencing was used to determine microbial community structures, followed by data mining of assemblies to identify AMR and pathogen incidence and enrichment across plastic types. To evaluate the importance of physical substrate, we studied colonization of four of the most common types of plastics found in the marine zone, namely polypropylene (PP), high-density polyethylene (HDPE), LDPE and PET [8, 17, 28, 29].

We hypothesised that: (A) the planktonic microbial communities in WWTP effluent, river, brackish and seawater will be distinct to those found in plastic associated biofilms. (B) The early-stage biofilm community on plastics will be distinct to the mature late-stage biofilm community structure. (C) Plastic will act as a vector for AMR-encoding wastewater-borne microorganisms, allowing them to persist after long term seawater exposure.

## Materials and methods

*Sampling location* 60 L of effluent (salinity 0.2, pH 7.6) wastewater was collected in October 2020 in acid-washed (10% phosphoric acid followed by distilled water) HDPE 20 L carboys directly from the effluent wastewater pipe at Llanrwst WWTP operated by Dŵr Cymru Welsh Water (53.13963, − 3.80351). An additional 60 L of Conwy River water (pH 7.3) was collected from a sampling site (53.14296, − 3.80700) downstream of the effluent pipe from the WWTP.

**Fig. 1:**
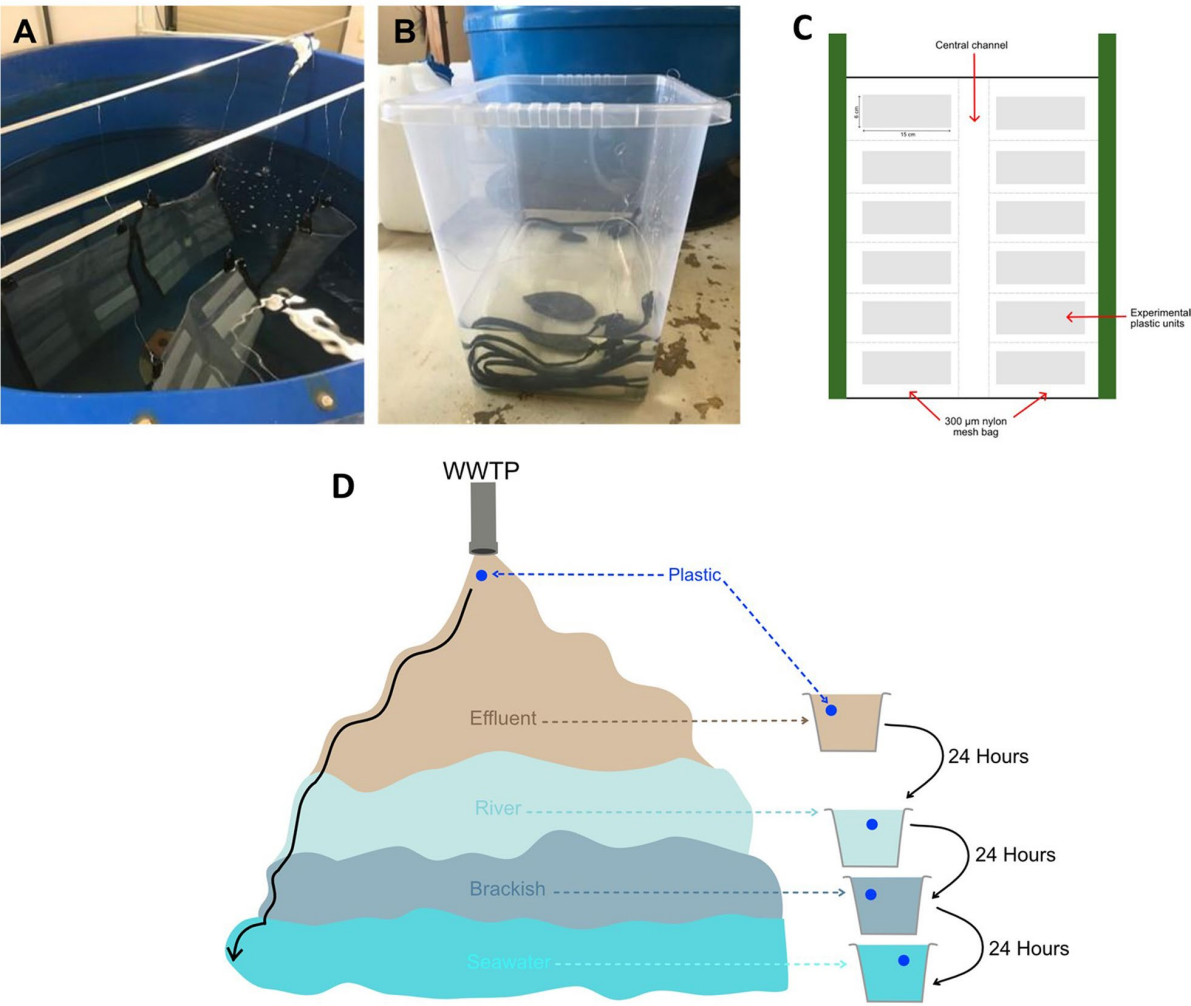
An overview of experimental setup. **A** One of the three mesocosm fibreglass tanks with suspended nylon mesh bags containing the plastic units. **B** One of the three storage containers used to carry out serial incubations, with river water incubation tank. **C** Nylon mesh bag layout. Each bag contained 12 experimental units of one plastic type (HDPE, LDPE, PET or PP). **D** Schematic of the experimental design where plastic was incubated in WWTP effluent, river water, brackish water and seawater to simulate the pathway plastic takes from WWTP to a marine environment

*Mesocosm tanks* Three independent 3 m^3^, indoor fiberglass tanks were used to carry out the controlled plastic exposures in the School of Ocean Sciences, Bangor University (Fig. 1). The tanks were filled with sand-filtered seawater pumped directly from the adjacent Menai Strait (53.21010, − 4.20262) and set up with a flow-through system allowing exchange of fresh seawater, and air traps to prevent sedentary conditions and to ensure oxygenation. An additional 350 µm filter was used to process water immediately before filling the tanks. Each tank was used as a technical replicate.

*Experimental set up* 1 mm thick plastic sheets of HDPE and PP and 0.1 mm thick plastic films of PET and virgin LDPE (Goodfellow, Cambridge, UK) were used to assess the formation of plastisphere communities. Films and sheets were cut into 15 × 6 cm experimental units. 300 µm aperture nylon mesh bags were used to contain the plastic experimental units used for the exposures. Each unit was held in place by its individual pocket (Fig. 1). A central channel in each bag was used to house Hobo Pendant® Temperature/Light 64K Data Loggers for monitoring temperature and light intensity. Each bag contained only one plastic type.

*Transition system incubations* A series of incubations were carried out to imitate the transition of the plastics through different water ecosystems. Nylon mesh bags containing the plastic experimental units were incubated in WWTP effluent for 24 h before being transferred to river water for 24 h. The river water incubation was followed by a brackish water incubation (1:1 mix of river water and mesocosm seawater) for 24 h, before being transferred to the seawater mesocosm tanks for the duration of the exposures. Above incubation times correspond to the typical transit times in the Conwy River headwater and brackish systems that ultimately feed into the Irish Sea [30, 31].

*Abiotic measurements* Hobo Pendant® data loggers were used to monitor irradiance and temperature through the transition system incubations and the seawater mesocosms over the course of the exposure experiment. At each sampling timepoint, salinity and pH measurements were recorded using a handheld refractometer and pH meter, respectively (Additional file 1: Table S1).

*Timepoints and sampling regime* At each incubation stage, 100 mL samples of WWTP effluent, river water and brackish water were taken in triplicate. For each seawater mesocosm tank, 1 experimental unit for each plastic type, a wall swab, and 300 mL of water were collected at each timepoint. Sampling timepoints were at 0-day, 1-day, 4-day, 1-week, 2-week, 3-week, 4-week, 6-week, 8-week, 12-week and 16-week. After collection, water samples were syringe-filtered through Sterivex® filters (pore diameter 0.22 µm, Merck Millipore, Darmstadt, Germany).

*Sample processing and storage* The plastic experimental units were aseptically cut into thirds (5 × 6 cm) and each segment placed into separate certified DNAse/RNAse free bags (VWR, Leicestershire, UK) and placed at – 20 °C for short-term storage (< 1 week).

*DNA extraction* All DNA extractions were carried out using the Quick-DNA Miniprep kit (ZYMO Research, Irvine, CA) according to the cell monolayer extraction protocol, with alterations to the cell lysis step for sample suitability as follows: lysis buffer was applied directly to samples in DNAse/RNAse free bags (VWR, Leicestershire, UK) and massaged to ensure complete coverage of the sample surfaces. Samples were left in lysis buffer for 10 min before the lysate was removed and processed in accordance with the manufacturer’s instructions. For the filtered water samples, the membranes were removed from the plastic Sterivex® filter casing and DNA extracted as described above. Extracted DNA concentrations were measured using Qubit dsDNA High Sensitivity Assay Kit on a Qubit 4 Fluorometer (Thermo Scientific, Oxford, UK). The yields of DNA varied in the range 100–300 ng, which was sufficient for the 16S rRNA gene V4 amplicon sequencing.

*16S rRNA gene amplicon sequencing and data analysis* The 16S rRNA gene amplicon sequencing was carried out for each sample (*n* = 3). In total, 207 samples were analysed. 16S rRNA gene amplicons were produced using duplicate amplification with double-indexed fusion primers as reported previously [32]. The hypervariable V4 16S rRNA gene region as PCR-amplified with modified forward primer F515 (5′-GTGBCAGCMGCCGCGGTAA-3′) and reverse R806 prokaryotic primer (5′-GGACTACHVGGGTWTCTAAT-3′) [32]. PCR was conducted using approx. 2 ng DNA template per reaction and OneTaq DNA Polymerase reagents (New England Biolabs, Ipswich, MA, USA). All PCR batches were run with no-template negative control samples. PCR conditions included: denaturation at 95 °C for 2 min with following 30 cycles of denaturation at 94 °C for 45 s, annealing at 55 °C for 1 min s, and DNA synthesis at 68 °C for 30 s with a final elongation at 68 °C for 5 min. PCR products were visualized in a 1.2% (TAE) tris–acetate-EDTA agarose gel using a GelDoc System (Bio-Rad, Hercules, CA). DNA bands of approximately 440 bp were gel-purified using the QIAEX II Gel Extraction Kit (Qiagen, Hilden, Germany). The purified amplicons were then quantified using the Qubit 4.0 Fluorometer, pooled in equimolar amounts and the final pool was run on Illumina MiSeq platform (Illumina Inc., San Diego, CA) using 500-cycle v2 chemistry (2 × 250 bp paired-end reads) at the Centre for Environmental Biotechnology, Bangor, UK.

Raw sequencing reads were processed according to previously described protocol [32, 33]. Briefly, the data was pre-processed to extract barcodes from the sequences, which were then cleaned of primer sequences using tagcleaner. The barcodes and the sequences were re-matched using in-house Python scripts. The resulting filtered reads were analysed using QIIME v2021.2 [34]. First, the libraries were demultiplexed based on the different barcodes. The sequences were then classified based on operational taxonomic units (OTUs) combining de novo and reference-based methods (open-reference OTU generation algorithm) using the SILVA version 132 reference database.

Analyses of most abundant taxonomic groups were performed using in-house R-based scripts, selecting those groups with a relative abundance of at least 2% in any of the samples. Selection started at genus level and groups were added to the immediate upper taxonomic level if none of the samples of that group passed the 2% threshold.

*Whole Genome Amplification (WGA)* As mentioned above, DNA yields amounted to 100–300 ng per sample. To produce sufficient DNA quantities for whole metagenome shotgun sequencing, WGA using a REPLI-g Mini Kit (QIAGEN, Hilden, Germany) was carried out according to the manufacturer’s instructions. Following amplification, total yields of DNA were between 0.45 and 6.0 µg per sample, which was sufficient for downstream Illumina NextSeq workflow. To test for potential biases in our DNA extraction, 16S rRNA amplification and WGA, we used ZymoBIOMICS Microbial Community Standard (ZYMO Research, Irvine, CA) alongside our experimental samples (Additional file 1: Fig. S1).

*Whole metagenome sequencing* Whole metagenome sequencing of 33 (Additional file 1: Table S2) samples selected, after the inspection of 16S rRNA gene amplicon sequencing data, was conducted externally at Novogene Ltd. (Cambridge, UK) using Illumina NovaSeq® paired-end (2 × 150) workflow to produce minimum 12 Gb of raw reads per sample.

*Metagenome assembly* BBtools software suite [35] was used to sort, filter and merge raw reads and the script repair.sh was used to separate unpaired and disordered reads. Contaminants and artefacts were then removed, and adapters and low-quality reads were trimmed using the script bbduk.sh. Filtered paired-end reads were merged using the script bbmerge.sh [36]. Final assembly was performed using SPADES genome assembler v3.15.4 [37, 38] with meta option for metagenomic data using merged and unmerged reads as input.

*Metagenome annotation and analysis* Assembled sequences were uploaded to IMG/M [39] for annotation under GOLD study ID Gs0154304. Metagenomes were compared using phylogenetic distribution with percentage identity of 90 + %. Abundance was normalised across all samples as hits returned per Mbp of assembled metagenome. Metagenome community structure bar charts were developed using R Statistical Software (v.4.2.2; [40]) using R packages, ggplot2 [41], tidyverse [42] and pals [43]. All taxa shown account for > 1% of the total community abundance to family taxonomic level. The community heatmap was produced using R packages gplots [44], tidyverse [42], and RColorBrewer [45], and were normalised by column.

*Antimicrobial resistance genes analysis* To study the content of antimicrobial resistance genes (ARGs) in our metagenomic samples, we had first to predict the protein coding genes from the assemblies produced in the step above. Prodigal 2.6.3 was used to find genes [46]. Subsequently, DeepARG 1.0.2 [47] was used to search for ARG in the coding gene sequences identified using Prodigal 2.6.3 for each metagenomic assembly. MetaCompare [48] was used to estimate the risk for ARG to be disseminated into human pathogens on environmental sample based metagenomic sequencing data. Average ARG abundance was calculated for each type of sample and normalized over the total gene abundance of each sample. Z-score, heatmap visualization and Resistome Risk graphic were developed using scripts on R environment [40]. All sequencing files have been deposited to the GenBank as BioProject PRJNA868775.

Additionally, ARG were identified in metagenomic data as shown in Additional file 1: Fig. S2. Polypeptide sequence similarity e-values cut off was taken at 1 × 10^–10^ with individually assigned bit score cut off according to the CARD database [49].

*Statistical analysis* Rarefaction was used to determine whether the 16S rRNA gene amplicon sequencing data had sufficient depth and coverage, as well as to determine if the depth of metagenome assembly was representative of observed sample richness to account for low whole genome assembly counts. Samples were rarefied individually as well as combined biological replicates to a subsampling threshold of the minimum number of gene counts in the data frame. Rarefaction was also used to assess diversity of ARG in each sample. Rarefactions analysed were produced using ggplot2 [41] and vegan [50] on R Statistical Software (v.4.2.2; [40]). Alpha diversity (Observed subsample species richness, Simpson diversity, inverse Simpson diversity and Shannon diversity) of raw 16S rRNA gene amplicon sequencing data were calculated using R Statistical Software (v.4.2.2; [40]). PERMANOVA statistical tests were used to determine significant interactions between timepoint and sample type in our 16S rRNA gene amplicon sequenced communities. This was followed by the Tukey post-hoc test, to determine the significant changes to community structures between timepoints. Additionally, PERMANOVA were used to assess the similarities and interactions of metagenome communities, AMR incidence, sample type and sampling timepoint, based on calculated Bray Curtis dissimilarity matrices. PERMANOVA results were used to produce non-metric multidimensional scaling (NMDS) plots and generated *p*-values were used to highlight significant interactions on heatmaps. All statistical analyses were carried out using R package vegan [50].

## Results and discussion

*16S rRNA gene amplicon sequencing and whole metagenome sequencing sample selection* As expected, mesocosm tank wall and seawater microbial communities remained more consistent over the course of the 16-week experiment, than the plastic communities that had been subjected to incubations in WWTP effluent, river and brackish water. Additionally, these samples proved to be less diverse than the plastic communities, having fewer taxa contributing less than 2% of their respective assembled sequences (Additional file 1: Figs. S3, S4).

**Fig. 2:**
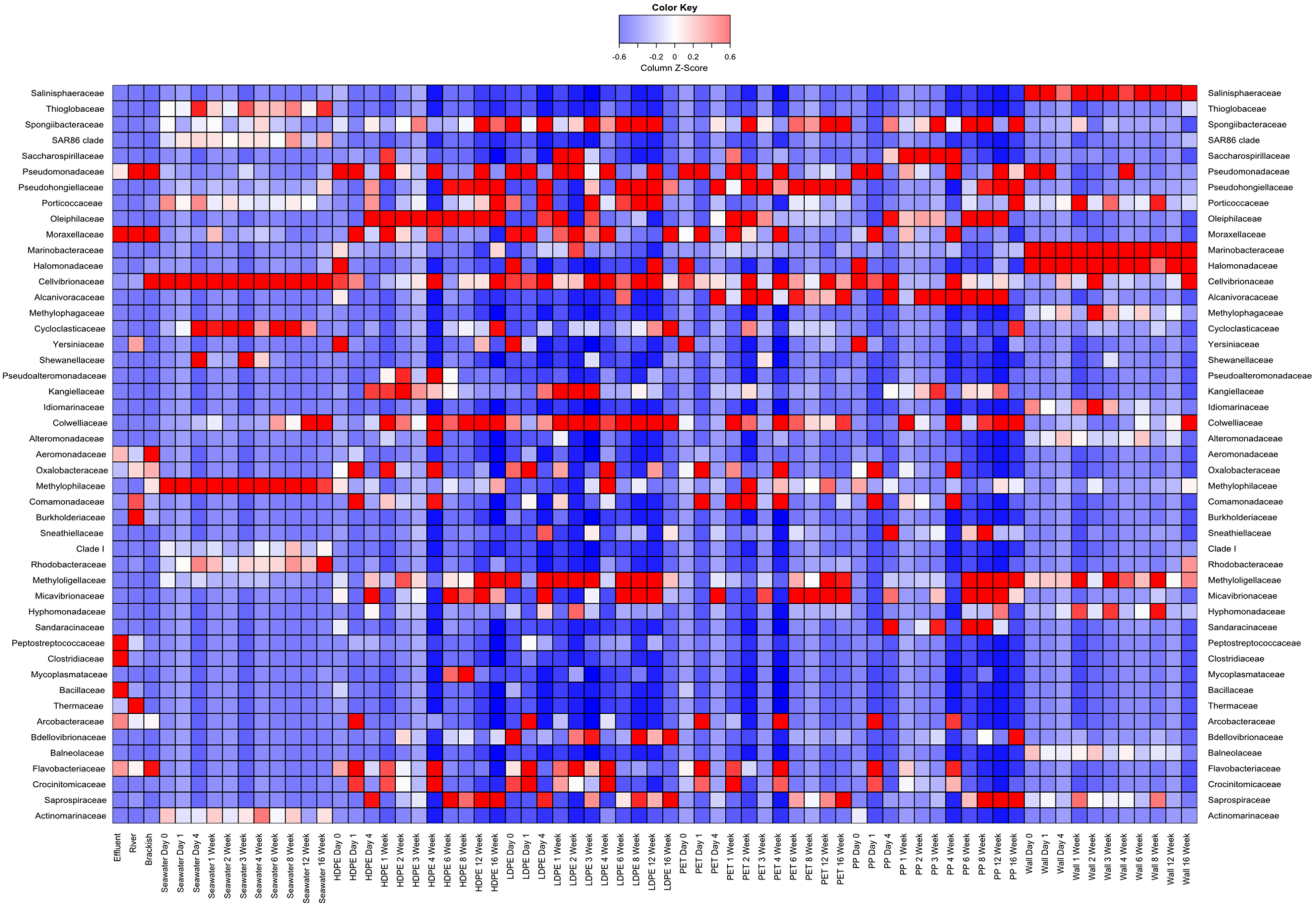
Heatmap of relative abundances of prokaryotic 16S rRNA gene amplicon reads. “Seawater Day 0—Seawater 16 Week” refer to the planktonic mesocosm seawater communities at corresponding timepoints. HDPE, LDPE, PET and PP refer to the compositions of plastic-associated biofilms that emerged after pre-incubation of above plastics in WW effluent (24 h), river (24 h) and brackish (24 h) waters and their exposure to the seawater in the mesocosms for periods between 0 days and 16 weeks. “Effluent”, “River” and “Brackish” correspond to planktonic microbial communities in fresh samples of WW effluent, river and brackish waters used for sequential pre-incubation of plastics. “Wall” samples correspond to microbial communities developing on the wall of mesocosm tanks

In all 16S rRNA gene amplicon data, *Pseudomonadota* was the dominant phylum. Overall, different sample types (water, plastic, tank wall) showed a distinct community structure (see Fig. 2 for an overview and, for a better resolution, in each sample type see Additional file 1: Figs. S3 and S4). Rarefaction curves of species richness (Additional file 1: Fig. S5) showed a tendency to saturation with the only exception of LDPE Week 12 as per rarefaction analysis. Baseline communities, i.e. wastewater effluent, river water, brackish water and planktonic microbial fraction of seawater, exhibited very distinct taxonomic compositions, with *Bacillaceae*, *Clostridiaceae* and *Peptostreptococcaceae* (combined, ca. 25% of all amplicon reads) predominant in the wastewater, were practically absent in all other natural samples.

Remarkably, a few taxa that were almost undetectable in seawater communities and absent in wastewater effluent, river water and brackish water, became selectively enriched on the polymeric materials. These included *Oleiphilaceae* who greatly increased their abundance from the early stages of colonisation on PET, LDPE, and even to a greater extent, throughout the whole time of residence on PP and HDPE in the seawater mesocosms. Among members of this family, it is well established that many are hydrocarbon degraders [51, 52], which may explain their enhanced affinity toward hydrophobic surfaces. Members of *Alcanivoracaceae* were significantly enriched on PET and PP. This family members include important and ubiquitous degraders of aliphatic hydrocarbons, weathered polyethylene and encode polyester-active enzymes [53–57]. Other taxa, such as *Saccharospirillum* [55], appeared on LDPE and HDPE for a short time, between weeks 1 and 3, and were abundant on PP between day 1 and week 4, however, after that their relative abundance reduced. *Saprospiraceae*, almost untraceable in baseline communities, became prominent in mature, late-colonisation-stage communities on all plastic types. Consistently with the large body of evidence, and as extensively reported earlier [55–57], these organisms are associated with “ixotrophic” predation of other bacteria. *Saprospiraceae* have previously been reported as a component of core plastic-colonising, truly marine, microbiomes [56–58].

Other predatory organisms, underrepresented in baseline communities, *Bdellovibrionaceae*, were seen in early-phase LDPE and HDPE-associated biofilms and in the very last week of PP colonisation. To conclude, we observed a rapid enrichment of ‘rare’ seawater taxa known for their ability to quickly colonise and occasionally degrade, polyolefins, which replaced the wastewater borne communities.

**Fig. 3:**
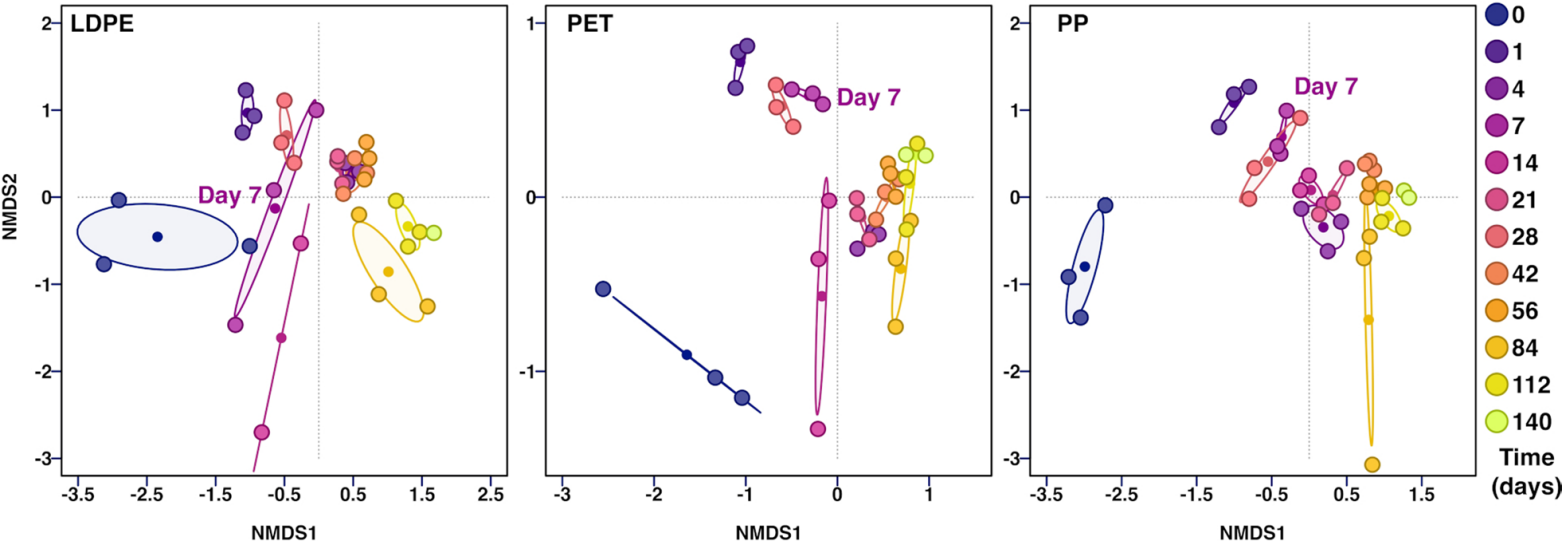
16S rRNA amplicon sequencing results Non-MultiDimensional Scale (NMDS) analysis for enrichments on LDPE, PET and PP for 140 days (20 weeks). Ellipses cluster samples of same timepoint. Major changes in the communities occur between timepoint 0 and 1 week, at the later colonisation stages, community compositions tend to co-cluster. This analysis has informed the selection of samples for shotgun sequencing

PERMANOVA statistical analysis of 16S rRNA amplicons suggested a wealth of significant interactions between bacterial families, sampling timepoints and sample types (Additional file 2: Table S3). This analysis guided the selection of DNA samples for whole metagenome sequencing. Metagenome sequencing provided a more focused examination of changes to community structure at the species level over time. It also enabled determination of ARG incidence and enrichment throughout the process. Additional file 1: Table S4 and Fig. 3 show that most of the significant changes to community structure took place within the first week of seawater exposure. Therefore, DNA samples from HDPE, LDPE, PET and PP 1-day and 1-week, along with effluent, river and mesocosm seawater baseline controls were selected for further whole metagenome shotgun sequencing for AMR gene analysis.

*Whole metagenome shotgun sequencing-resolved microbial communities* Sequencing data outputs varied between samples and treatments (Additional file 1: Tables S2 and Additional file 2: Table S5). Combined rarefaction curves showed assembled metagenomes for the HDPE treatment did not meet the minimal richness threshold (Additional file 1: Fig. S6). We have therefore not included HDPE 1-day and HDPE 1-week samples into the subsequent analyses. As expected, for the remaining samples, LDPE, PET, and PP, microbial diversity was higher after 1-week of incubation compared to 1-day. Alpha diversity indices for metagenomic assemblies (Additional file 2: Table S6) pointed at an overall higher diversity in 1-day samples as compared with 1-week, with a broader range of low-abundance prokaryotes (< 1% of the assembled community) (Additional file 1: Fig. S7 and Additional file 2: Table S6). PERMANOVA tests showed significant interactions between certain bacterial families and sample types. The most significant interactions (*p* < 0.05) were for the plastic-colonising communities after 1-week of incubation. Due to low coverage and low gene counts for PET and PP 1-day samples, we excluded those from our analyses and focused on changes between the baseline communities (effluent, river and seawater) and plastic samples exposed for 1-week.

**Fig. 4:**
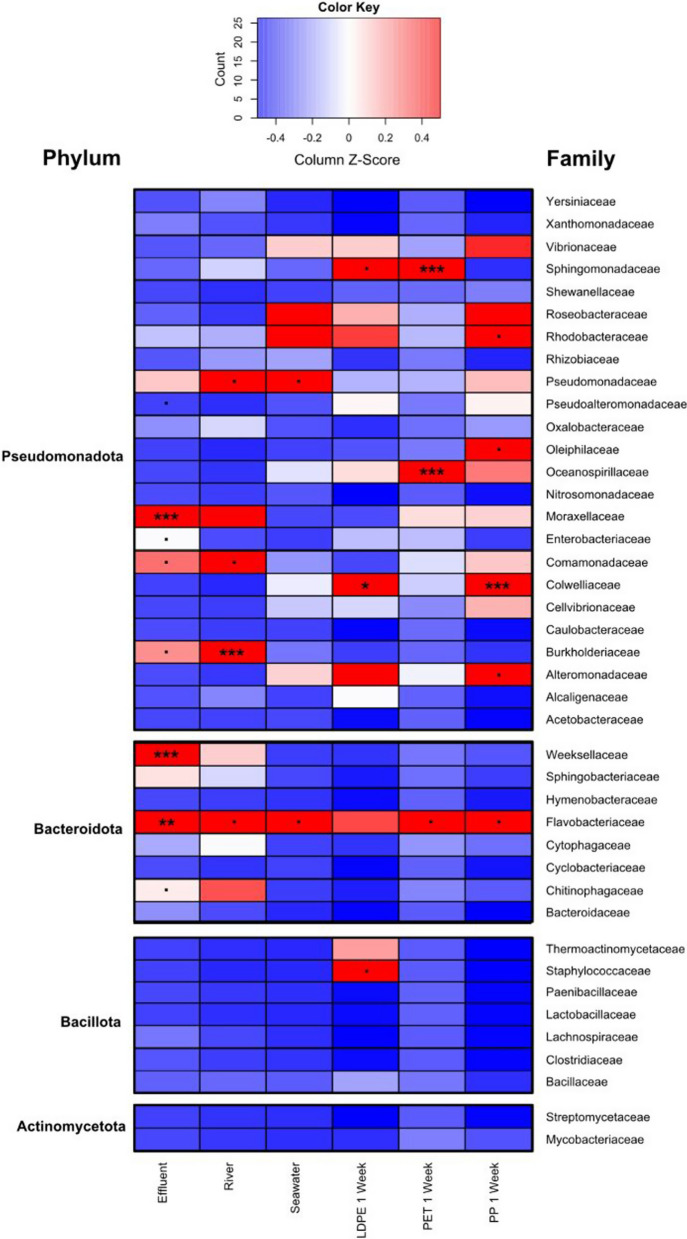
Relative abundance of bacterial families in microbial communities from different water and plastic surface samples based on number of reads mapped per million base pairs (Mbp) of assembled metagenome. Samples include wastewater treatment plant (WWTP) effluent, river water, seawater, and biofilms on low-density polyethylene (LDPE), polyethylene terephthalate (PET), and polypropylene (PP) plastics. Plastics were incubated sequentially in WWTP effluent, river water, brackish water, and seawater for 1 week each prior to sampling. Protein sequence identity cut off at 90% + . ANOVA-generated *p*-values for interactions between sample type and bacterial family are shown on significant heatmap tiles. Statistical significance: ***p < 0.001; **p < 0.01; *p < 0.05; *p* < 0.1

Following determination of reliable and suitable shotgun sequenced samples, NMDS was conducted to compare whole metagenome data to 16S rRNA gene amplicon community data (Additional file 1: Fig. S8). Although the two approaches showed some differences in community structures (Additional file 1: Figs. S3, S4, S9), the overall pattern remained the same. WWTP effluent and river water samples exhibited structures distinct from those from plastic samples. The communities of plastic colonisers showed higher similarities with seawater after just 1 week of incubation, pointing at a rapid replacement of wastewater- and river water-associated microbes on the plastic surfaces with marine microorganisms (Fig. 4).

**Fig. 5:**
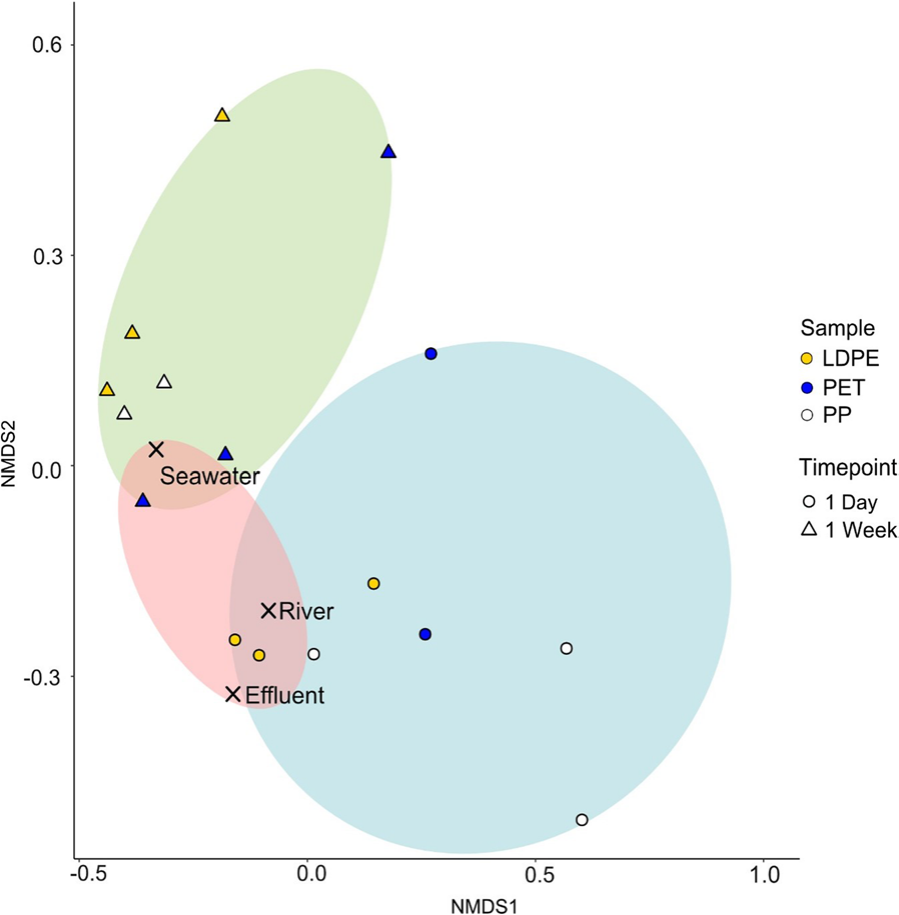
Bray Curtis based non-metric multidimensional scaling plot of plastisphere sample community structure following successive incubations of plastic in WWTP effluent, river water, brackish water and seawater from WGS data. Centroids of baseline communities (planktonic WWTP effluent, river water and seawater) are labelled. Coloured bubbles represent the areas covered by each timepoint. Timepoints of 1-day and 1-week are shown by different shapes and plastics (LDPE, PET and PP) by colour

PERMANOVA analysis of the microbial metagenomes showed distinct clustering patterns for all of our samples. Figure 5 shows plastic microbial communities after 1-week co-clustered with those of seawater, whereas 1-day plastic communities tend to overlap with those of the WWTP effluent and river water samples. This points at the rapid, within one week, replacement of WWTP effluent microorganisms with the seawater microbiome. Microbial diversity was higher in plastic biofilm communities than in planktonic communities, with more taxa contributing < 1% of the total metagenome [59].

At a family level, all plastic types supported development of unique bacterial community structures with statistically significant interactions between microbial taxa and sample type, as shown in Fig. 4. At a phylum level, communities were dominated by *Pseudomonadota* followed by *Bacteriodota*, which is consistent with previous 16S rRNA gene amplicon-based studies on plastic associated biofilms and wastewater [17, 59–62]. Like in those studies, statistical tests offered a clear clustering at early timepoints which become dispersed at later timepoints as mature biofilms formed [18].

Across all samples, *Flavobacteriaceae* was the only microbial family to be consistently found throughout the experiment and in significant amounts for WWTP effluent, river and seawater as well as plastic biofilms. *Flavobacteriaceae* appear to be ubiquitous in freshwater and marine environments and are secondary biofilm colonisers due to their adaptability to abiotic changes and potential to use extracellular polymetric substances (EPS) excreted by primary colonisers as an energy source [63]. Our metagenomic data showed an abundance of *Rhodobacteraceae* in seawater, LDPE 1-week and significant amounts in PP 1-week. *Rhodobacteraceae* produce EPS and may help in formation of early conditioning biofilms [60, 61]. Along with being common marine microorganisms, this could explain why *Rhodobacteraceae* remain abundant on plastic surfaces at 1-week incubation point. Our metagenomic data also appears to show an increase in *Alteromonadaceae* abundance following WWTP effluent and river water incubations, consistently with previous studies showing their key role in early biofilm formation [60].

For the majority of metagenomes, seawater, LDPE 1-week, PET 1-week and PP 1-week differed significantly in microbial composition from WWTP effluent and river water communities. Previous research showed that plastisphere communities are influenced to a greater extent by environmental variables than substratum types [9]. Our metagenomic data supports this hypothesis, with common seawater microorganisms dominating plastic community structures after 1 week exposure to the seawater. However, microbial taxa and their abundance do not remain consistent across all plastic types (e.g., *Sphingomonadaceae*, *Oceanospirillaceae*, *Colwelliaceae*) suggesting their selective preference for certain substratum characteristics. This may also relate to different chemical additives present within the plastics [56, 60]. Distinct differences between WWTP effluent, river and seawater metagenomes allowed us to determine how primary plastic-colonising communities become structured and influenced by different water bodies and how certain microbes preferentially accumulate on plastic surfaces (Fig. 4). Among the taxa underrepresented in metagenomes of baseline communities of wastewater, river- and seawater, few were significantly (orders of magnitude) enriched on specific plastic surfaces (Additional file 1: Fig. S9) and have been categorised as Most Abundant Taxonomic Groups (MATGs). Members of hydrocarbon-degrading genus *Oleibacter*, which was recently [64] merged with the genus *Thalassolituus,* the type strain of which was originally described by Yakimov et al. [65] as an obligate hydrocarbonoclastic bacterium with a high affinity to hydrophobic compounds, were predominant (> 10%) taxa on PET, which was also supported by 16S rRNA amplicon sequencing (Additional file 1: Fig. S9B). Methylotrophic alphaproteobacteria of the order *Rhodobacterales, Marinibacterium* spp. [66], were strongly enriched on PP, while many taxa abundant in baseline communities, e.g. *Pelagibacter* and BACL14 clade both overrepresented in the seawater and *Polynucleobacter* abundant in the river water, were present on plastic surfaces in insignificant numbers being replaced by taxa discussed above that have a higher affinity to polymeric surfaces.

**Fig. 6:**
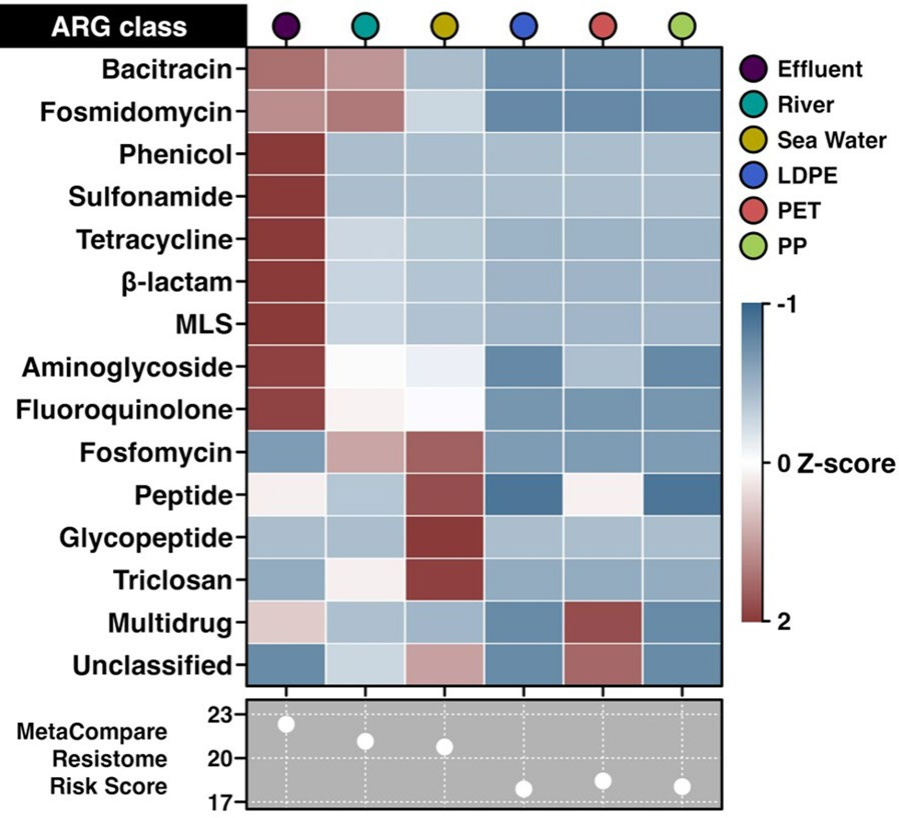
Relative abundance of ARG classes across different microbial communities within wastewater treatment plant effluent, river water, seawater, and microbial communities colonising PET, PP and PE after successive passage through these water types and 1 week in seawater mescocosm. Z-score has been calculated by row, based on the DeepARG deep-learning model

*AMR gene incidence in metagenomes* The metagenome DeepARG search revealed that the relative abundance of ARGs was highest in WWTP effluent, which also consequently had a highest Resistome Risk Score following Metacompare analysis [47]. All plastic-colonising microbial communities had a lower incidence of ARG and lower Resistome Risk Score in comparison to WWTP effluent, river water and seawater. Overall, ARG abundance followed the order WWTP effluent > river water > seawater > PET > PP and LDPE (Fig. 6). This suggests that under given conditions, the tested plastic species did not become enriched for microorganisms harbouring principal ARG classes. This is consistent with the shotgun metagenomic analysis data presented above, indicating the replacement of initial effluent-borne organisms by typical seawater taxa.

**Fig. 7:**
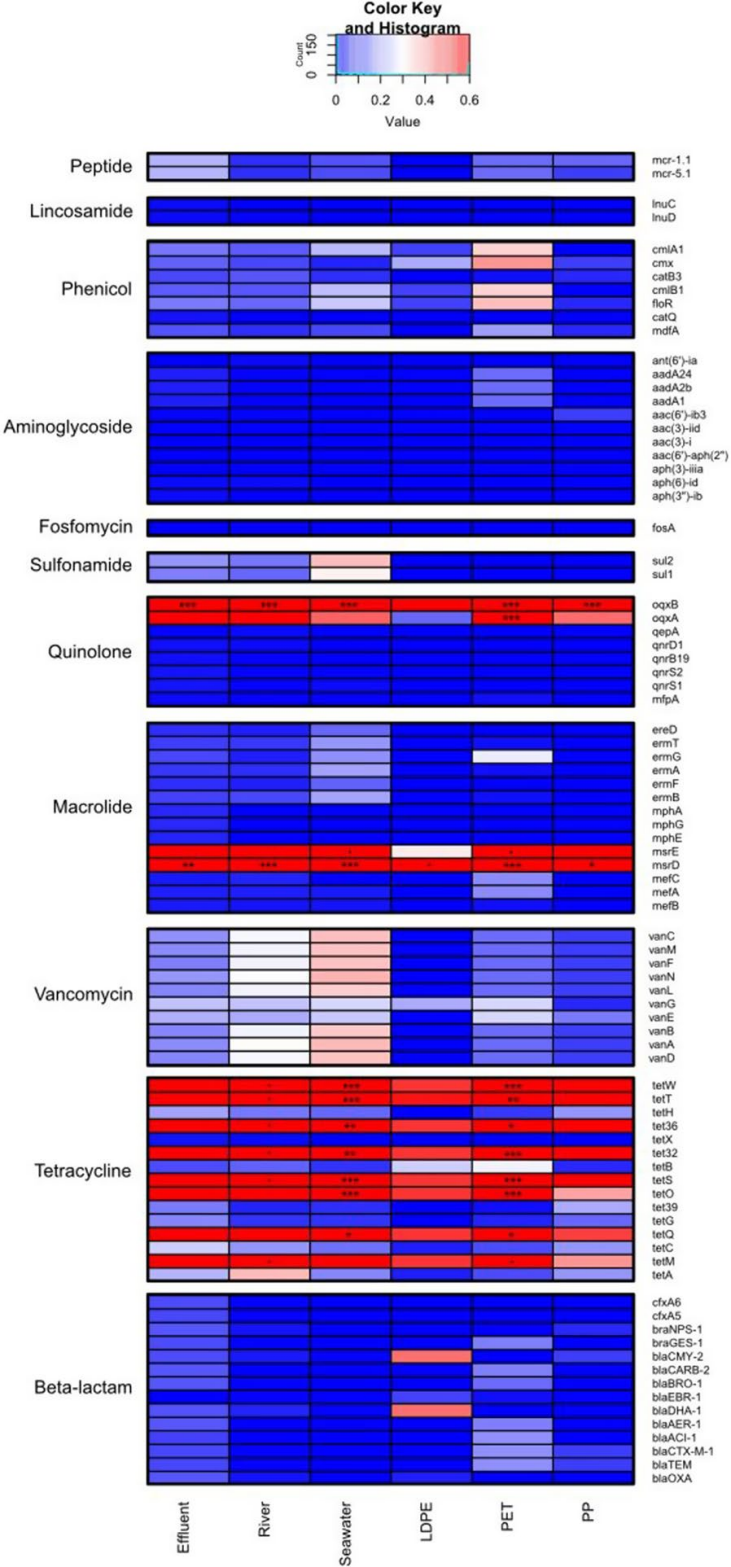
AMR gene copy number per Mbp of assembled metagenomes normalised for microbial communities in wastewater treatment plant effluent, river water, seawater, and communities that colonise LDPE, PET and PP plastics after pre-exposure to wastewater, river water brackish water and seawater at 1-week. Abundance changes are shown by the colour gradient. Significant ANOVA generated *p*-values for interactions between sample type and ARG are shown on significant heatmap tiles. Statistical significance: ***p < 0.001; **p < 0.01; *p < 0.05, *p* < 0.1. Based on CARD database [49]

Furthermore, baseline communities (WWTP effluent, river, seawater) and plastic-colonising microbiomes were scored against the 100 ARGs that are the most-abundant in WWTP effluent samples from 101 countries [27, 67] (Fig. 7). This analysis showed that although ARG did persist on plastic surfaces, their abundance did not increase over time. Outliers to this observation were *oqxA* and *oqxB*, which exhibited an increase between 1-day and 1-week. Originally found on the chromosome of *Klebsiella pneumonia*e conferring resistance to quinolone antibiotics (as well as some detergents, disinfectants, and other antimicrobials) via efflux pump, these genetic loci have also been detected on mobile genetic elements (MGE) [68], which, as we discuss below is not the case.

Our results indicated a high abundance of tetracycline class ARGs across all samples. Tetracycline class antibiotics are typically persistent in the environment and maintain their active form following use [69]. In Wales, this class of antibiotic are one of the most commonly prescribed in healthcare [67, 70]. Additionally, tetracycline is regularly used in aquaculture and livestock farming. Tetracycline resistance genes accounted for 17% of the ARG identified in this study. Statistically significant copy numbers of resistance genes *tetQ*, *tetO*, *tetS*, *tet32*, *tet36*, *tetT* and *tetW* were found in seawater samples and in the PP biofilm at 1-day, and PET biofilm after 1-week. *tetM* was also abundant in seawater, with *tetH* readily found in the PP biofilm on 1-day. Many of these *tet* genes are associated with MGE which could explain their persistence within the metagenomic data across the course of the study [49]. However, this does not explain the lack of enrichment of these genes in the plastic biofilms and seawater after 1-week.

Macrolides are another class of the most clinically prescribed antibiotics in the study area and make up significant proportion of our AMR profile [70]. Macrolides accounted for 16% of the total ARG count. Statistically, *msrD* was the most abundant gene for macrolide class of ARGs, with a high abundance in WWTP effluent, river water and seawater, and the LDPE biofilm after 1-day, and PET and PP biofilms after 1-week. The ARGs *msrD* (chromosomally encoded) and *msrE* (plasmid-associated) appear to persist over the course of the experiment in contrast to the other macrolide ARGs investigated, which remained low in gene counts and therefore may not be as mobile [49].

As mentioned above, quinolones, a broad-spectrum class of synthetic antibiotics, accounted for a large proportion of the AMR genes across all samples. This was unsurprising given previous reports of their prevalence in WWTP effluents [27]. The genes encoding OqxA and OqxB components of an efflux pump, were found to have high copy numbers in the baseline (WWTP effluent, river water and seawater) samples, as well as in the LDPE biofilm after 1-day and PET and PP biofilms after 1-week [27, 49]. *oqxA* and *oqxB* were the most persistently occurring quinolone resistance genes found in our study. As quinolone antibiotic use has been decreasing in Wales since the withdrawal of the ‘4C antimicrobials’ [67, 70], and close monitoring of quinolone in primary care was introduced to prevent the transmission of AMR and prevent broad spectrum antibiotics becoming ineffective [70], the plausible reason for their consistent detection across all samples in present study is that above multidrug efflux pumps/transporters from RND (Resistance-Nodulation-Division) family are naturally occurring in practically all genomes of free-living, seawater and freshwater bacteria. Indeed, the deduced OqxAB were not MGE-, but chromosomally-encoded, and had significant amino acid sequence identities with counterparts from RND multidrug efflux pump proteins from common non-pathogenic bacteria.

β-lactam associated ARGs also accounted for a large proportion (16%) of the AMR profile in our study. Following incubation in WWTP effluent, β-lactam class ARGs appeared to persist in biofilms on the different plastics, as opposed to their planktonic counterparts. Statistically significant gene counts of *blaTEM*, and *blaCTX-M-1* were detected in PP biofilms after 1-day alongside *blaACL-1*, *blaAER-1*, *blaBRO-1*, *blaCARB-2* and *blaGES-1*. These genes are class A β-lactamases (serine proteases) and are the most commonly detected class in WWTP effluents [71, 72] .

*AMR gene incidence: effects of abiotic factors and and sampling location* Our findings of AMR classes abundances were consistent with those reported by Javvadi and Mohan [62], with large proportions accounted for loci for resistance to by tetracyclines, β-lactams and MLSBs (macrolides). Additionally, “Universal ARG”, *mphE*, *msrE*, *tetA*, *tetC*, *tetW*, *sul1* and *sul2* were detected in wastewater samples, consistently with Munk et al. [27], although not all of them persisted on plastic surfaces, or even after river- and seawater incubations. This is consistent with previous studies showing differences in AMR profiles in response to geographical location, antibiotic use, wastewater treatment method and a range of biotic and abiotic factors [18, 24–26, 69]. Some of the antibiotics discussed are persistent in the environment, or excreted as active compounds that act as selection pressures encouraging the acquisition of ARG [16, 20]. Llanrwst WWTP serves a population of around 4000 people in a largely agricultural area where effluent wastewater is discharged directly into the river following filter bed secondary treatment [73]. Abiotic factors, such as salinity and temperature in WWTP were earlier reported of being important in AMR profiles [18, 24–26, 69]. Warmer temperatures provide optimal conditions for bacterial growth and HGT, hence seasonal changes have an impact on AMR profiles, with higher ARG loads in spring than in winter [74]. Counts of tetracycline, sulfonamide and vancomycin classes of ARG tend to be significantly higher in winter [69]. As our sampling campaign took place in the winter, these abiotic factors may have had a notable effect on AMR profiles reported in this study.

## Conclusions

In this study, mesocosm experiments were conducted to simulate the transition of plastic materials, PE, PET and PP, from wastewater treatment plant through the river into the sea, and to assess changes in microbial community compositions of plastic-colonising microorganisms and their AMR gene repertoire. Clear distinctions between communities in WWTP effluent, river water, brackish water and seawater and those colonising plastics were observed. Plastic surfaces preferentially selected taxa typical in marine systems, many of which are known for polyester and hydrocarbon degrading activities, but did not support waste- or freshwater-borne organisms. Predatory bacterial taxa were also detected with *Saprospira* spp. feeding on mature communities. Some ARG classes retained their relative abundances during seawater exposure experiments, however, those were affiliated with the innate gene repertoire of common seawater microorganisms, which generally pose a lower health risk.

### Supplementary Information

**Additional file 1**. It contains Supplementary Figures S1-S9 and Supplementary Tables S1, S2 and S4

**Additional file 2**. It contains Supplementary Tables S3, S5 and S6
