# Supplementary material for: Microbial communities colonising plastics during transition from the wastewater treatment plant to marine waters
Source: Environ Microbiome. 2024 Apr 29;19:27. doi: 10.1186/s40793-024-00569-2 (PMC11057073; doi:10.1186/s40793-024-00569-2)
Supplement: Supplementary file 1 — Additional file 1. It contains Supplementary Figures S1-S9 and Supplementary Tables S1, S2 and S4 [file 40793_2024_569_MOESM1_ESM.docx]

**Supplementary information to the paper of**

**Tulloch, C.L. et al.,** Temporal dynamics of microbial communities colonising plastics during transition from the wastewater treatment plant to marine waters

**Supplementary Figures S1-S9** **Pg. 2-13**

**Supplementary Tables S1, S2, S4**  **Pg. 12-14**

**Supplementary Tables S3, S5 and S6 are in the separate Excel spreadsheet file**

**References Pg. 15**


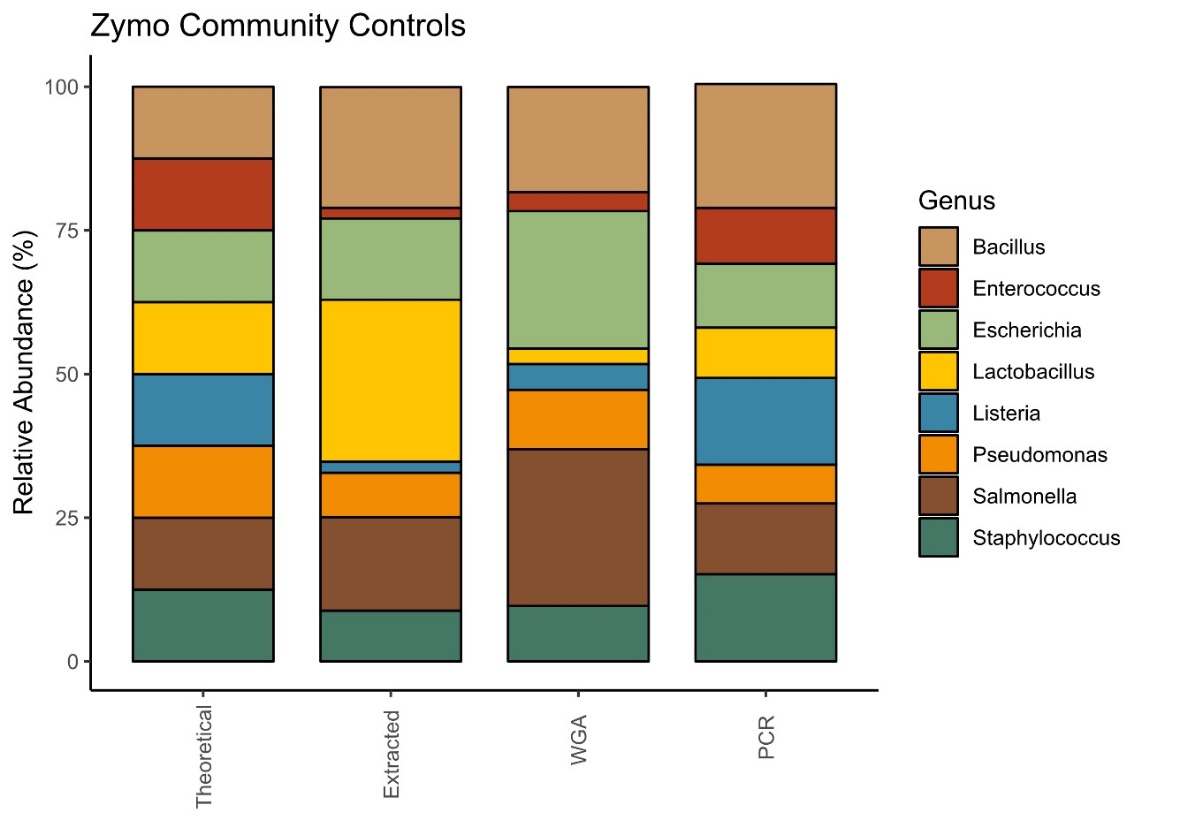


**Figure S1.** Assessment of 16S rRNA gene amplicon sequencing data of ZymoBIOMICS Microbial Community Standard (ZYMO Research, Irvine, CA) after DNA extraction with Quick DNA Extraction Kit (ZYMO research) and whole genome amplification (WGA) using REPLI-g Mini Kit (QIAGEN, Hilden, Germany) used in this study. Fungal microbial content is not shown. Theoretical abundance refers to the expected yield based on the mock microbial community composition in the ZYMO standard (‘Theoretical”). ‘PCR’ refers to the results of PCR amplification directly after DNA extraction and amplicon sequencing data analysis; ‘WGA’, to amplicon sequencing data after the whole genome amplification of extracted DNA and 16S rRNA gene PCR amplification.


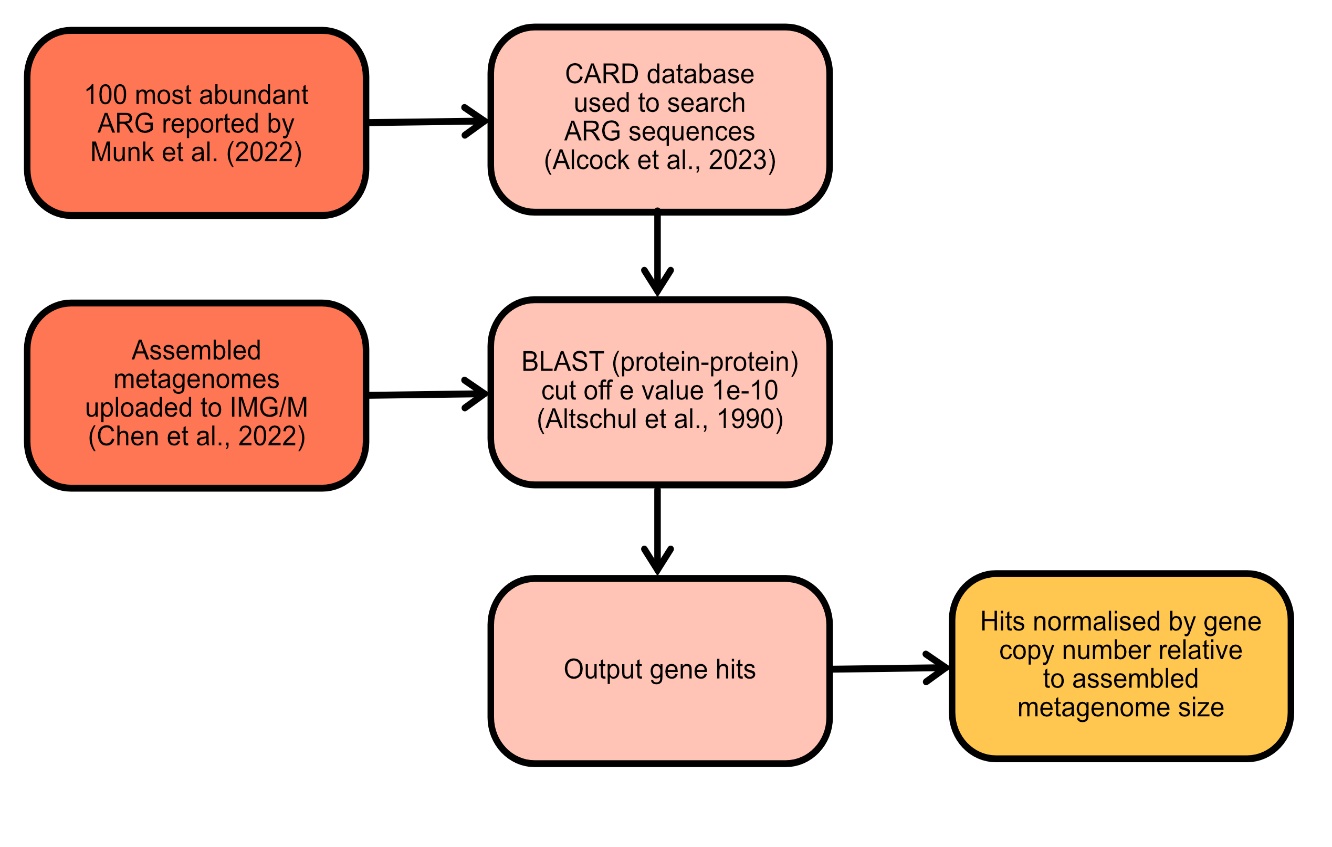


**Figure S2**. The bioinformatics workflow for prediction and enumeration of ARGs present in the metagenomic dataset.



**Figure S3.** Relative abundance of bacterial families from 16S rRNA amplicon sequencing in “baseline” planktonic communities of WWTP effluent, river and brackish waters. Families of the same phylum are grouped by colour. Families with >2% of total reads are shown. “Minor taxa” refer to families with less than 2 % reads, or unclassified taxa.


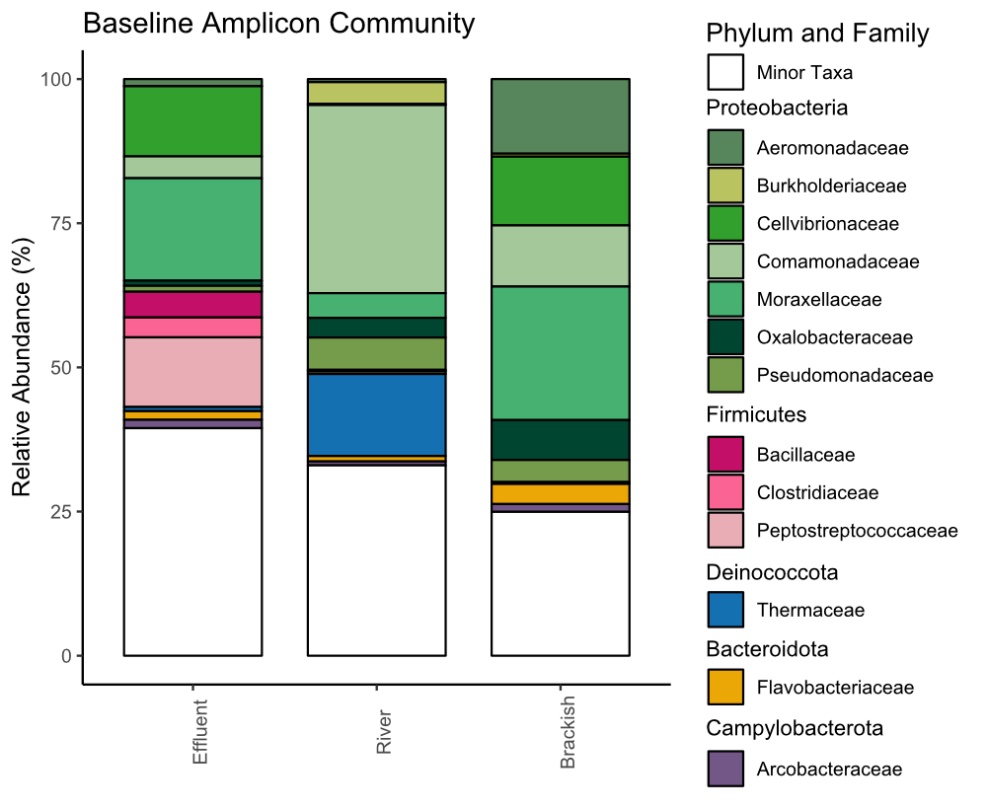

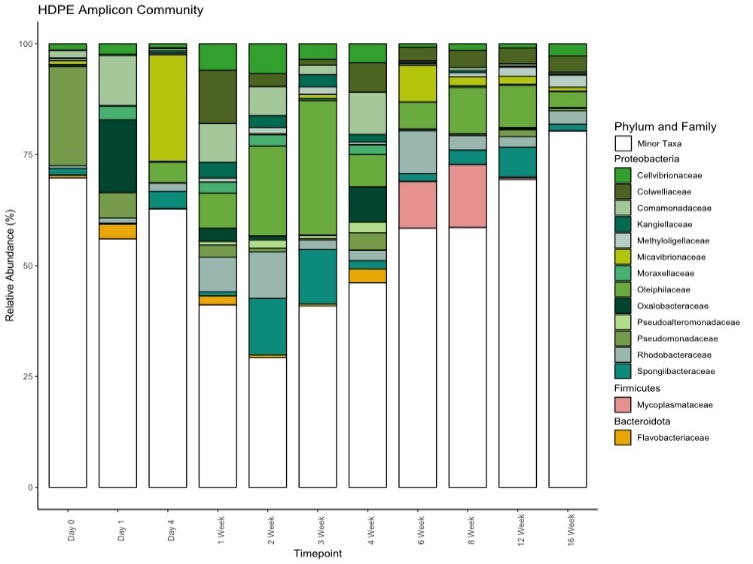

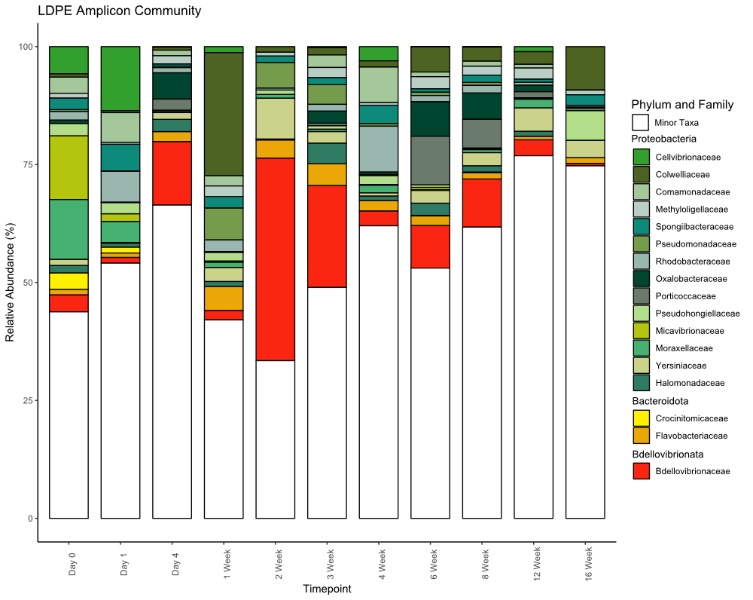

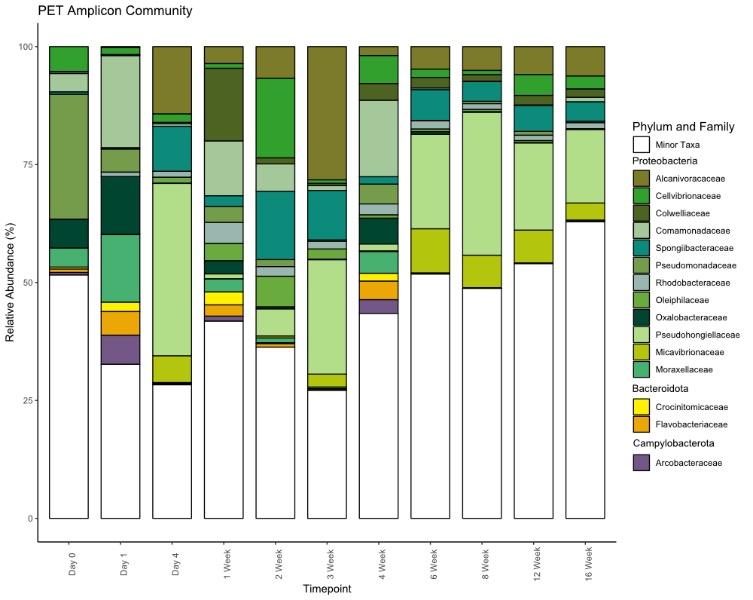

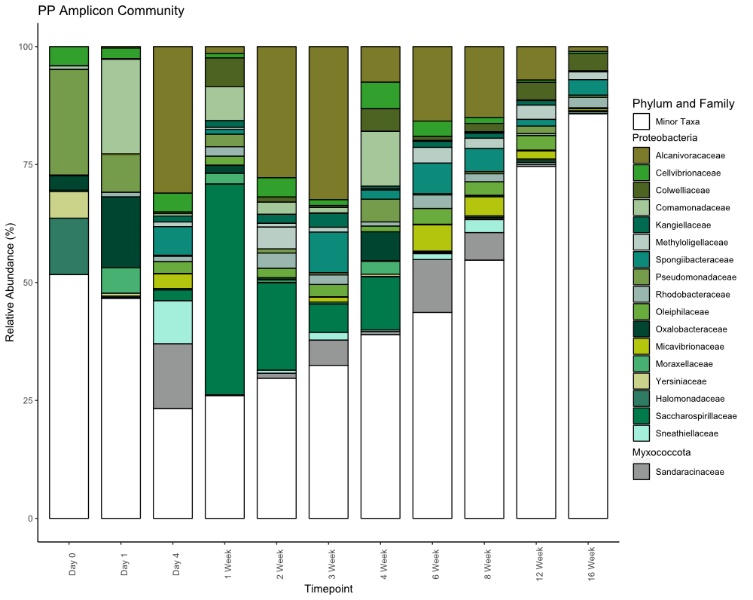

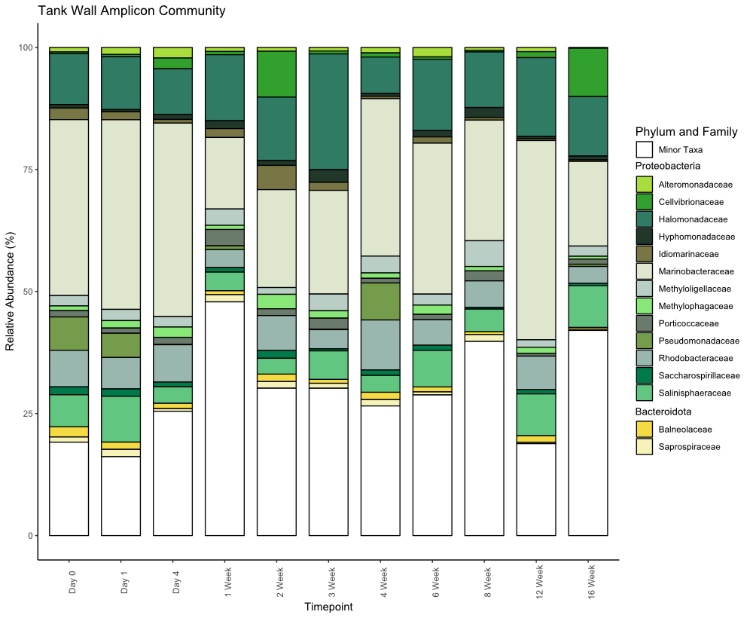

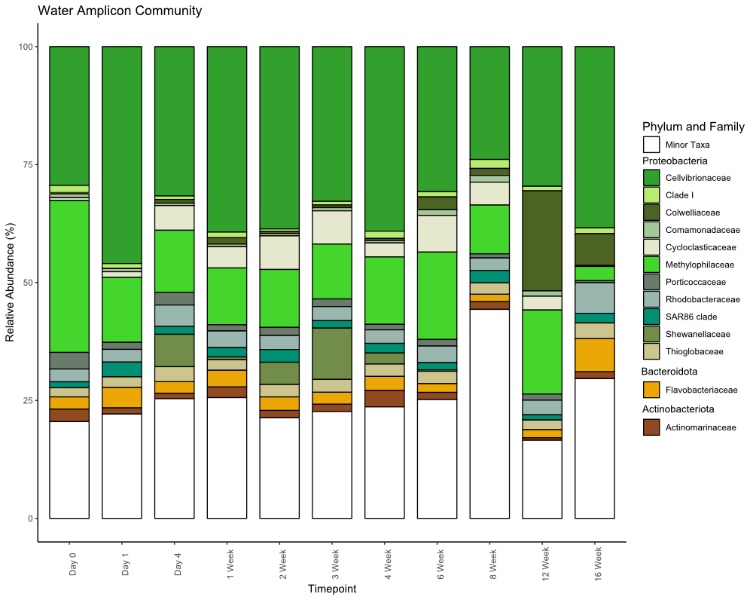


**Figure S4.** Bar charts showing relative abundance of bacterial families from 16S rRNA amplicon sequencing. HDPE, LDPE, PET and PP communities refer to plastic-associated biofilms at each timepoint (0-day to 16-week) of their residence in seawater mesocosms, after pre-incubation in: WWTP effluent (24 h), river (24 h), and brackish (24 h) waters. ‘Tank wall’ communities are those sampled from mesocosm tank wall throughout the experiment and ‘Seawater communities’ refer to planktonic mesocosm seawater communities at corresponding time points. Families from the same phyla are grouped by colour. Families making up 2% or more of amplicon reads are shown. A) HDPE community, B) LDPE community, C) PET community, D) PP community, E) Tank wall community, F) Mesocosm seawater planktonic community. **‘**Minor taxa’ refer to families with <2 % of reads, or unclassified taxa.

**A**

**B**

**C**

**D**

**E**

**F**


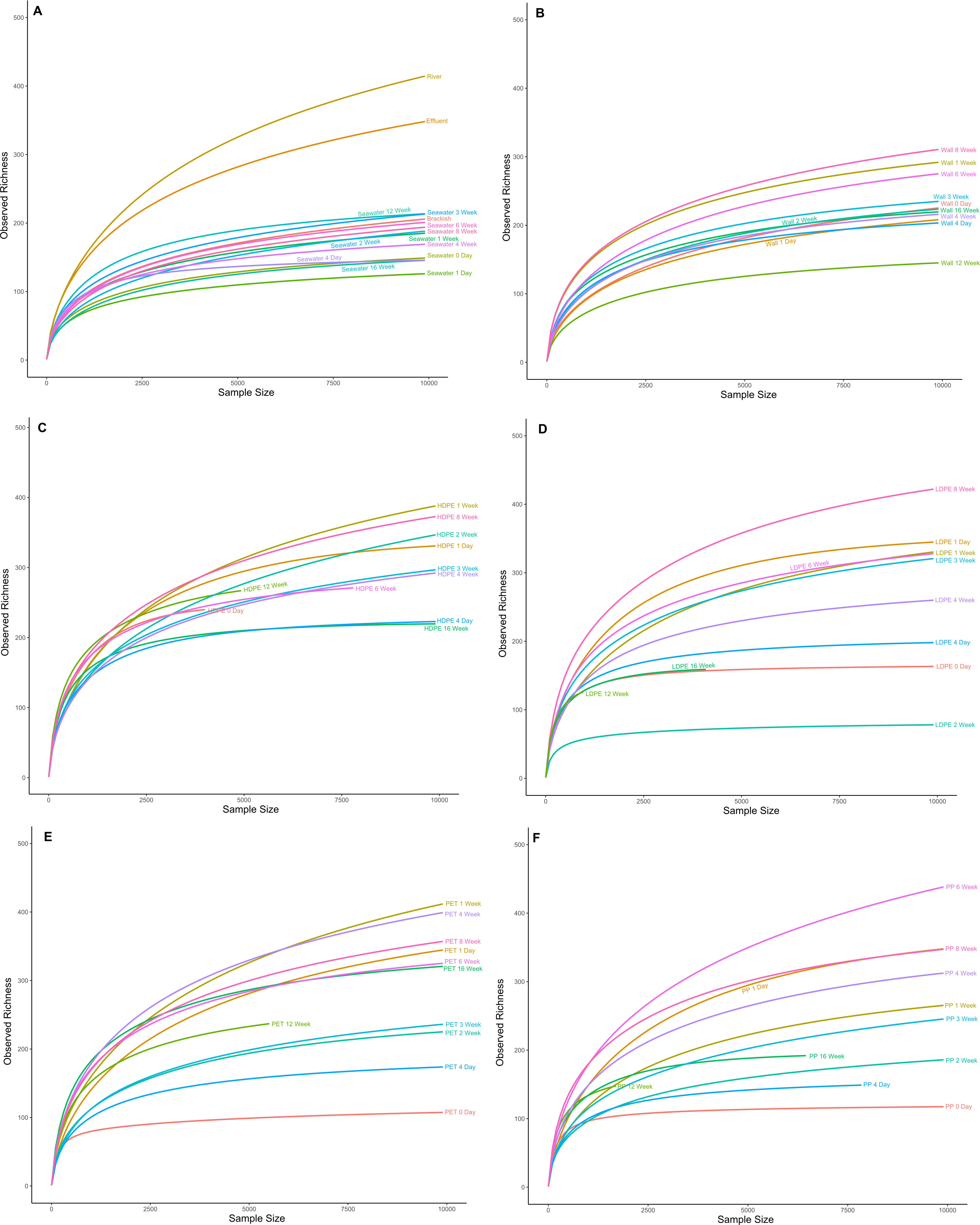


**Figure S5.** Rarefaction curves of 16S rRNA amplicon sequencing richness across all timepoints. Sample replicates were combined. Sample size refers to the number of sequences per sample with cut off at n = 10,000. HDPE, LDPE, PET and PP communities refer to plastic-associated biofilms at each timepoint (0-day to 16-week) of their residence in seawater mesocosms, after pre-incubation in: WWTP effluent (24 h), river (24 h), and brackish (24 h) waters. WWTP effluent, river, brackish data refer to amplicon reads from planktonic water samples at each incubation step. Tank wall rarefactions are based on sequencing reads from mesocosm tank wall swabs taken throughout the 16-week experiment. A) planktonic seawater and baseline communities , B) Tank wall, C) HDPE, D) LDPE, E) PET, F) PP.

**Figure S6.** Rarefaction curves of metagenome richness obtained from whole metagenome sequencing data. HDPE, LDPE, PET and PP plastic-associated biofilms sampled after incubation in the seawater mesocosm at 1-day and 1-week, after their pre-incubation in WWTP effluent (24 h), river (24 h) brackish (24h) waters. WWTP effluent, river water and seawater planktonic communities are also shown. The dashed red line indicates the subsample size used for comparison. A) Rarefaction curves of individual samples. B) Rarefaction curves of combined replicates for each type of samples**.**


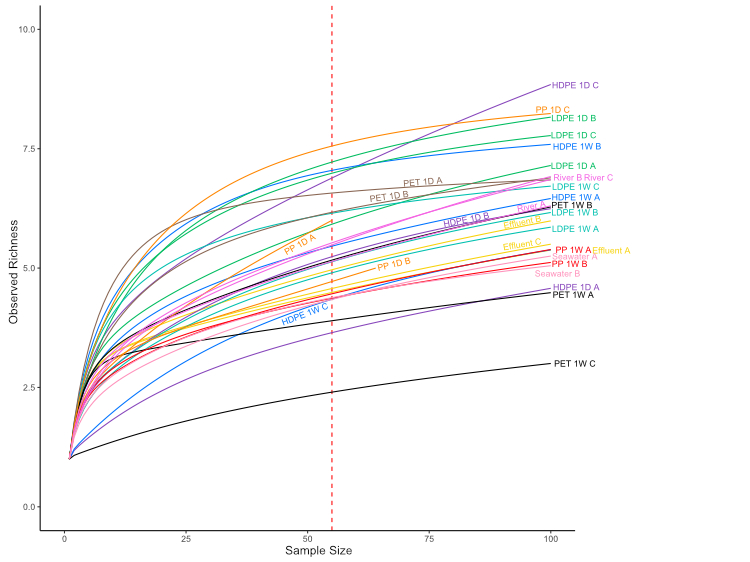

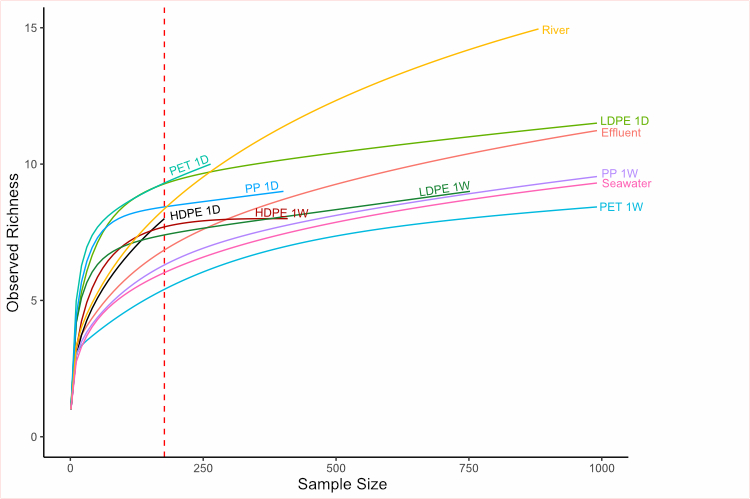


**A**

**B**

**Figure S7.** Community bar charts of bacterial families per Mbp of assembled metagenome. Identity cut off 90+ %. Organisms with abundance >1 % of the metagenome are shown. A) Baseline planktonic communities of wastewater treatment plant effluent, river and seawater. B) Plastic-associated communities at sampling timepoints 1-day and 1-week following incubations in WWTP effluent, river, brackish and seawater. Minor taxa refer to those making up <1 % of the metagenome or those that are unclassified.


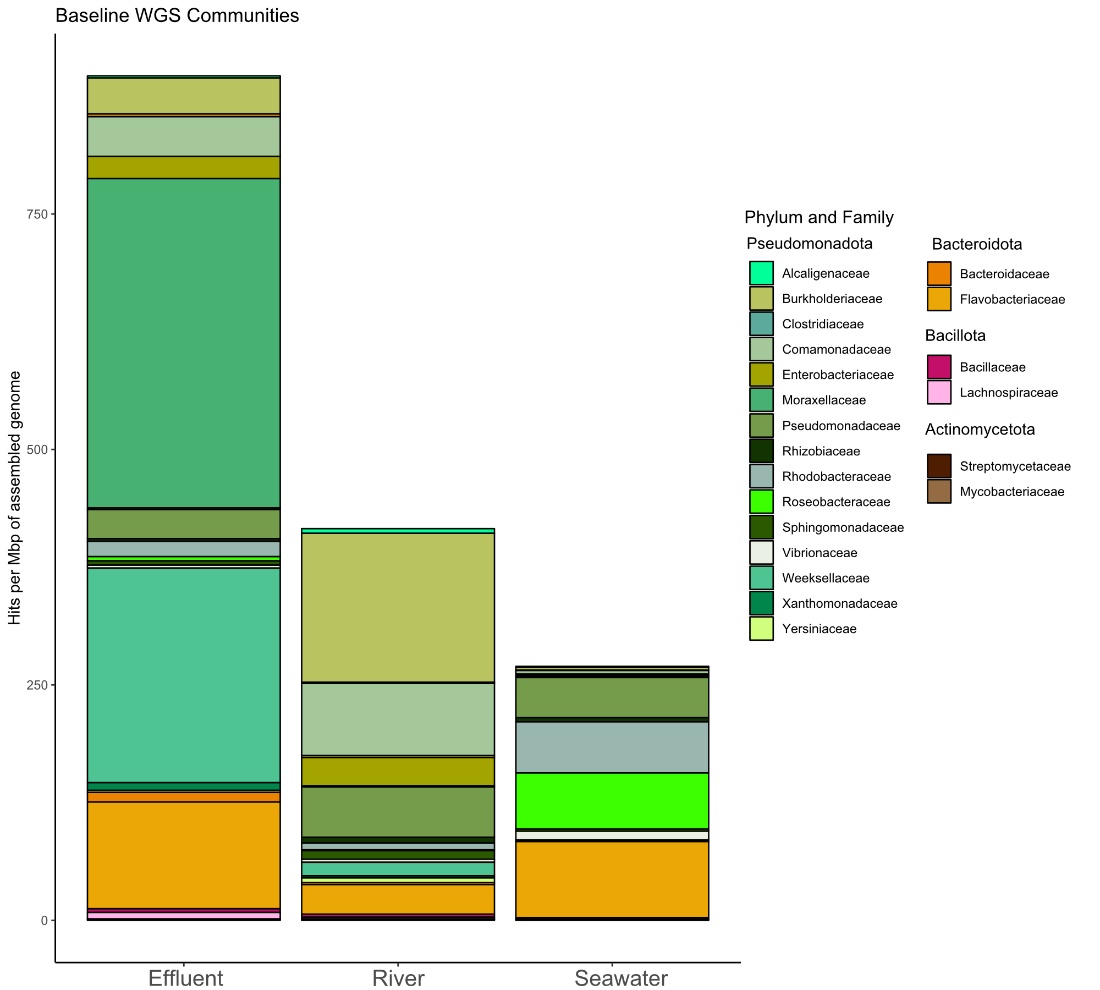

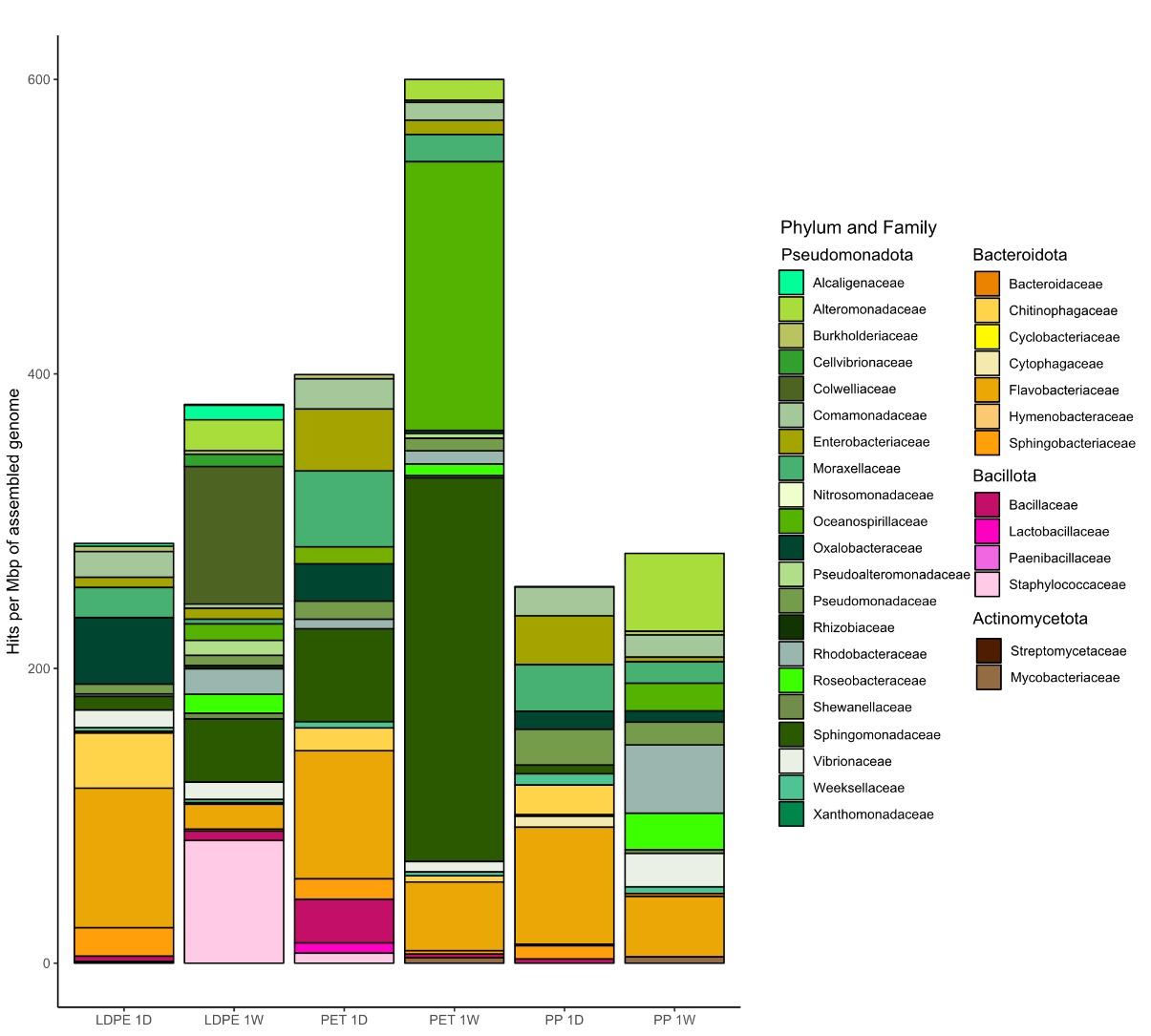


**A**

**B**

**Figure S8.** Comparative non-metric multidimensional scaling plot of WGS and 16S rRNA gene amplicon-based analyses. Coloured points represent sample types and shapes the method of sequencing. WWTP effluent, river water and seawater community data are shown. Plastic associated biofilms on LDPE, PET and PP after 1 week exposure to seawater after preliminary incubation in WWTP effluent, river, brackish and seawater are also shown.


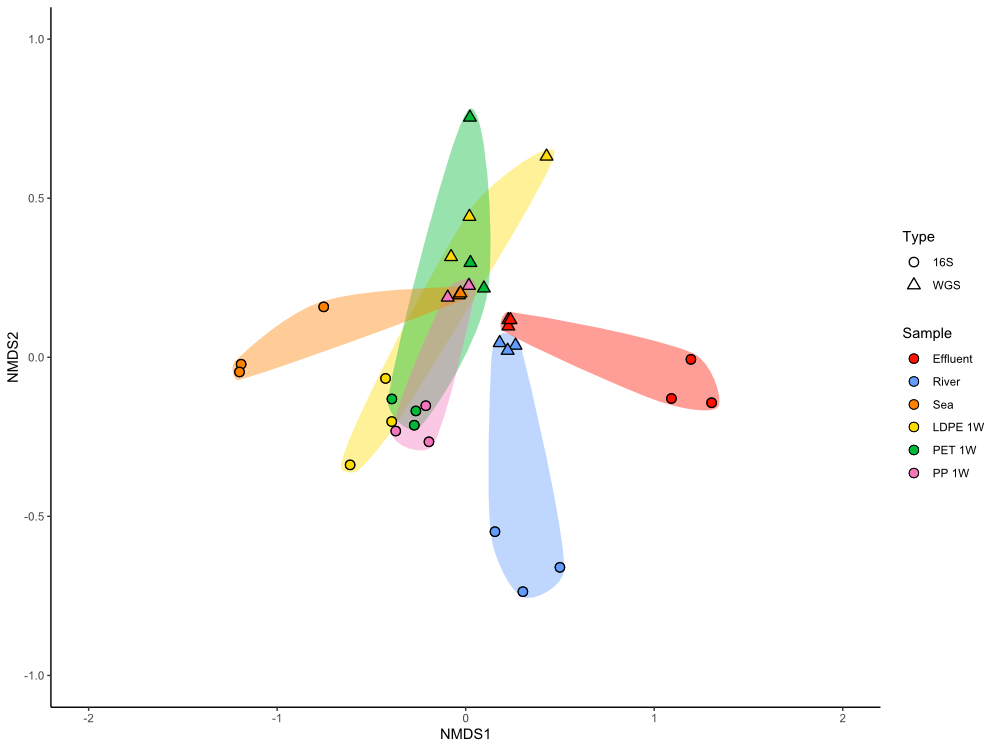


A


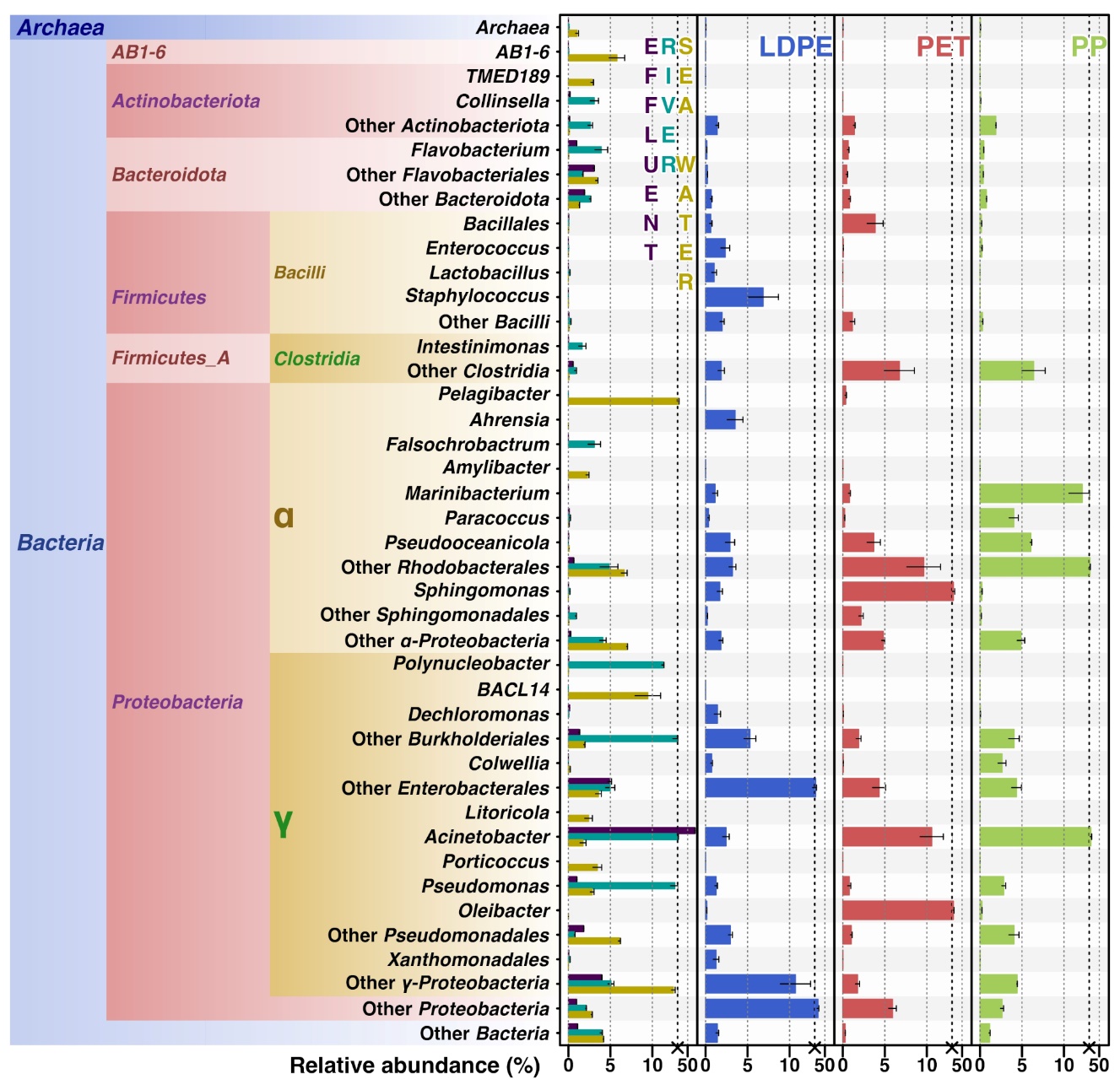


B


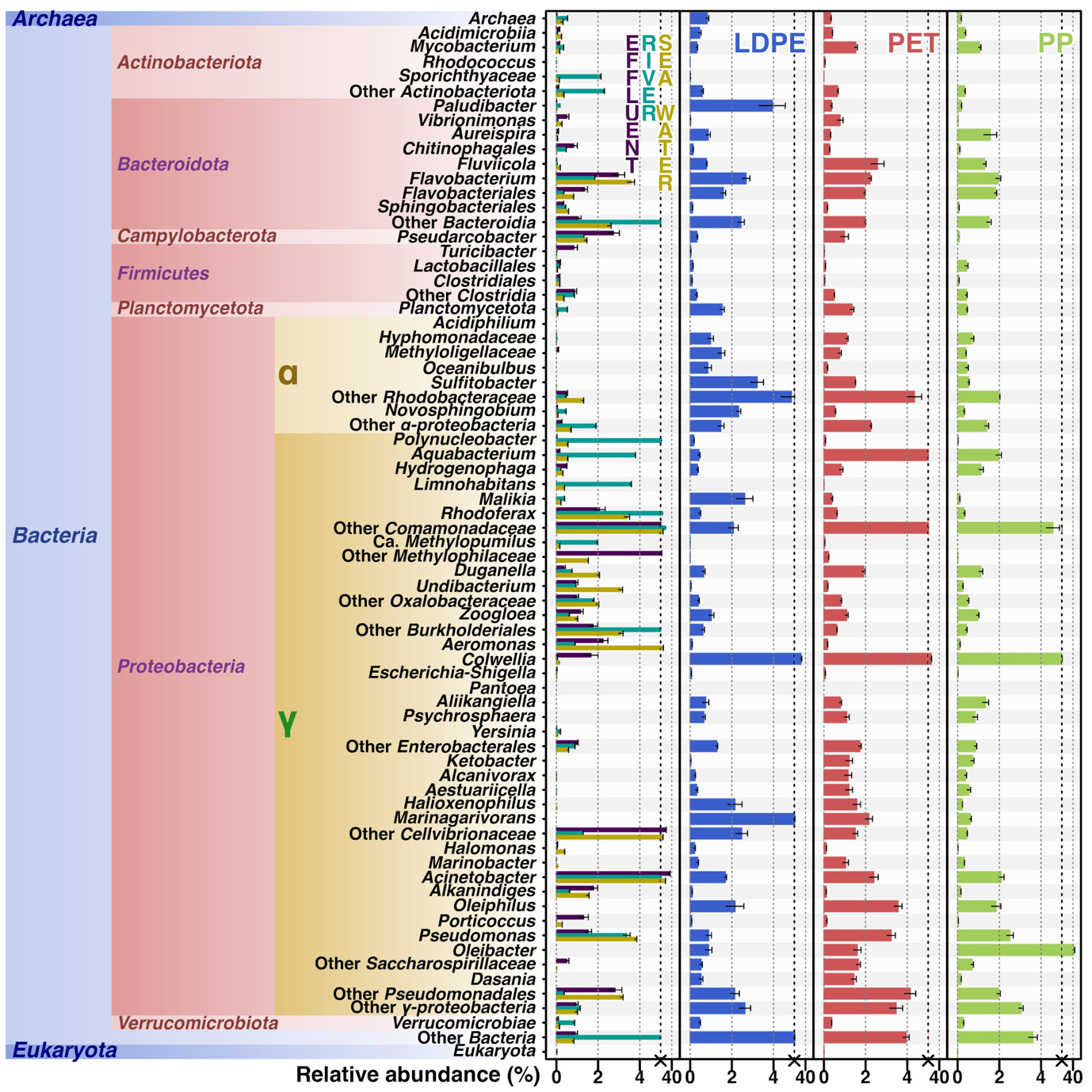
**Figure S9**. Relative abundance of Most Abundant Taxonomic Groups (MATGs) based on metagenomic (A) and amplicon (B) sequences with 0.02 confidence in the baseline microbial communities (‘Effluent’, ‘River’ and ‘Seawater’) and on plastic surfaces of PP, PET and LDPE after one week of incubation in the seawater mesocosm. MATGs were considered when a taxon had above 3% of affiliated metagenomic reads (A) and 2% 16S rRNA gene amplicon (B). Taxonomic assignment has been performed using Kraken v2.0.8-beta based on GTDB (Genome Taxonomy Database) (A) and Silva v.138.

**Table S1**.Abiotic conditions measurements across the course of the experiment. a, b, c refer to replicate tanks. WWTP effluent, river water and brackish mix refer to the initial incubation stages. 0-day to 16-weeks to mesocosm seawater samples**.**

**Table S2**. Summary table of whole genome sequencing assemblies. A, B and C refer to sample replicates.

**Separate Excel file:**

**Table S3**. Summary table of PERMANOVA statistical significance. Results are shown for interactions between family, sample and timepoint in the 16S amplicon communities.

**Table S5**. Summary table of PERMANOVA statistical significance. Results are shown for interactions between ARG and sample type in the WGS communities.

**Table S6.** Alpha diversity indices of microbial taxa predicted in metagenomic assemblies.

**Table S4**. Numbers of significant changes to biofilm communities on HDPE, LDPE PET and PP following incubations in WWTP effluent, river, brackish and seawater between each timepoint from 16S rRNA gene amplicon sequencing at genus taxonomic level. The number of changes was determined using a Tukey post hoc statistical test. Confidence level, *p*<0.05.

**References**

Alcock, B. P., Huynh, W., Chalil, R., Smith, K. W., Raphenya, A. R., Wlodarski, M. A., Edalatmand, A., Petkau, A., Syed, S. A., Tsang, K. K., Baker, S. J. C., Dave, M., McCarthy, M. C., Mukiri, K. M., Nasir, J. A., Golbon, B., Imtiaz, H., Jiang, X., Kaur, K., McArthur, A. G. (2023). CARD 2023: expanded curation, support for machine learning, and resistome prediction at the Comprehensive Antibiotic Resistance Database. Nucleic Acids Research, 51(D1), D690–D699. https://doi.org/10.1093/nar/gkac920

Altschul, S. F., Gish, W., Miller, W., Myers, E. W., & Lipman, D. J. (1990). Basic Local Alignment Search Tool. In J. Mol. Biol (215).

Chen, I. M. A., Chu, K., Palaniappan, K., Ratner, A., Huang, J., Huntemann, M., Hajek, P., Ritter, S. J., Webb, C., Wu, D., Varghese, N. J., Reddy, T. B. K., Mukherjee, S., Ovchinnikova, G., Nolan, M., Seshadri, R., Roux, S., Visel, A., Woyke, T., Ivanova, N. N. (2023). The IMG/M data management and analysis system v.7: content updates and new features. *Nucleic Acids Research*, 51(1 D), D723–D732. https://doi.org/10.1093/nar/gkac976

Munk, P., Brinch, C., Møller, F. D., Petersen, T. N., Hendriksen, R. S., Seyfarth, A. M., Kjeldgaard, J. S., Svendsen, C. A., van Bunnik, B., Berglund, F., Bego, A., Power, P., Rees, C., Lambrinidis, D., Neilson, E. H. J., Gibb, K., Coventry, K., Collignon, P., Cassar, S., Aarestrup, F. M. (2022). Genomic analysis of sewage from 101 countries reveals global landscape of antimicrobial resistance. Nature Communications, 13(1). https://doi.org/10.1038/s41467-022-34312-7
